# Supplementary material for: Management of children visiting the emergency department during out-of-office hours: an observational study
Source: BMJ Paediatr Open. 2020 Sep 15;4(1):e000687. doi: 10.1136/bmjpo-2020-000687 (PMC7493126; doi:10.1136/bmjpo-2020-000687)
Supplement: Supplementary data [file bmjpo-2020-000687supp001.pdf]

SUPPLEMENTARY APPENDIX

Supplement 1. Presenting problems

Supplement 2. Immediate Life-saving Interventions

Supplement 3. Missing data for vital signs

Supplement 4. Complete case analyses

Supplement 5. Hospital characteristics

Supplement 6. Stratified regression analyses

Schinkelshoek G, *et al. BMJ Paediatrics Open* 2020; 4:e000687. doi: 10.1136/bmjpo-2020-000687

Supplement 1. Presenting problems

Supplementary table 1.1 Presenting problems definitions

**Subgroups**[a]	**MTS Flowchart**
**General malaise**	Crying baby. Unwell child. Worried parent. Irritable child. Unwell adult.
**Trauma or muscular**	Assault. Falls. Head injury. Limb problems. Major trauma. Torso injury. Neck pain. Back pain. Limping child.
**Gastro-intestinal**	Abdominal pain in adults. Abdominal pain in children. Diarrhoea and vomiting. GI Bleeding.
**Respiratory**	Asthma. Shortness of breath in adults. Shortness of breath in children.
**Eear, Nose, Throat**	Ear problems. Sore throat. Facial problems.
**Neurologic or Psychiatric**	Behaving strangely. Fits. Headache. Apparently drunk. Collapsed adult. Overdose and poisoning. Self-harm. Mental illness.
**Dermatologic**	Bites and stings. Burns and scalds. Wounds. Abscesses and local infections. Rashes.
**Uro- or Gynaecological**	Testicular pain. Urinary problems. Sexually acquired infection. Pregnancy. PV Bleeding.
**Cardiac**	Chest pain. Palpitations.
**Other**	Allergy. Dental problems. Diabetes. Exposure to chemicals. Eye problems. Foreign body. Major incidents – primary. Major incidents – secondary. General/other.

[a]Subgroups of presenting problems (MTS Flowchart), based on a previous study (1)

Supplementary table 1.2 Presenting problems

N (%)	**Office hours (n=50,417)**	**Evening shift (n=36,429)**	**Night shift (n=14,138)**	**Day shift weekend (n=18,220)**
**General malaise**	10,069 (20.0)	7,021 (19.3)	3,473 (24.6)	3,784 (20.8)
**Trauma or muscular**	9,785 (19.4)	7,281 (20.0)	1,161 (8.2)	3,111 (17.1)
**Gastro-intestinal**	7,613 (15.1)	5,274 (14.5)	2,860 (20.2)	2,470 (13.6)
**Dermatologic**	5,864 (11.6)	4,851 (13.3)	1,044 (7.4)	2,864 (15.7)
**Respiratory**	5,686 (11.3)	3,833 (10.5)	2,494 (17.6)	1,947 (10.7)
**Ear, Nose, Throat**	4,777 (9.5)	3,596 (9.9)	1,320 (9.3)	1,870 (10.3)
**Neurologic or Psychiatric**	2,115 (4.2)	1,384 (3.8)	628 (4.4)	566 (3.1)
**Uro- or Gynaecological**	1,126 (2.2)	800 (2.2)	240 (1.7)	416 (2.3)
**Cardiac**	635 (1.3)	438 (1.2)	177 (1.3)	148 (0.8)
**Other**	2,084 (4.1)	1,489 (4.1)	443 (3.1)	733 (4.0)
**Missing**	690 (1.4)	462 (1.3)	298 (2.1)	311 (1.7)

Supplement 2. Immediate life-saving interventions

Supplementary table 2. Immediate life-saving interventions[a]
1. Airway and breathing support, including intubation or emergent non-invasive positive pressure ventilation.
2. Electrical therapy, including defibrillation, emergent cardioversion, or external pacing.
3. Procedures, including chest needle decompression, pericardiocentesis, or open thoracotomy.
4. Hemodynamic support, including significant intravenous fluid resuscitation in the setting of hypotension, blood administration, or control of major bleeding.
5. Emergency medications, including naloxone, dextrose, atropine, adenosine, epinephrine, or vasopressors

[a]Adapted from Lee et. al.(2)

Schinkelshoek G, *et al. BMJ Paediatrics Open* 2020; 4:e000687. doi: 10.1136/bmjpo-2020-000687

Supplement 3. Missing data for vital signs

The missing data for the vital signs variables (heart rate 42.7%, respiratory rate 52.4%, oxygen saturation 44.9%, temperature 22.5%) were assumed to be missing at random. We expected that the pattern of missingness could be explained by different patient and setting-related factors, that were included in the dataset. For example, based on clinical experience, measuring vital signs was considered less useful or relevant in low urgency cases or in patients with specific presenting problems, such as simple fractures. Also in high urgency cases data of vital signs was missing, which was not expected. This could be partly explained by data of vital signs that were not documented in the patient record, instead of vital signs that were not measured. The proportion of missing data for vital signs was comparable with previous studies. (3-5) A variety in the proportion of missingness was observed between the different hospitals, which is likely due to differences in characteristics of the patient populations and in local policies and compliance to these policies. (Supplementary figure 1)
In exploratory analyses, strong associations were found between the proportion of missing data and several patient and hospital-related factors (type of presenting complaint, triage urgency and disposition after the ED visit), thereby supporting the assumption that the missing data for vital signs were missing at random.
The missing data were imputed using a multiple imputation model including general patient characteristics, date and time of visit, triage items, vital signs, diagnostics, therapy and disposition. (Supplementary table 3)
Imputation was performed by using the MICE imputation package in R, version 2.15.2. The imputation process resulted in twenty-five datasets on which statistical analyses were performed and pooled for a final result.

Supplementary table 3. Multiple imputation model

Type of information	Variables
**General patient characteristics**	Hospital (cat); Age (cont.); Gender (M/F); Referral type (Self / GP / Emergency services / Other)
**Date and time**	Arrival month (1-12); Arrival hour (morning/evening/night); Arrival day (week/weekend)
**Triage items**	MTS flowchart (cat); MTS urgency (1-5); MTS painscore (cont)
**Physiological parameters**	Heart rate (cont); Respiratory rate (cont); Temperature (cont); Systolic bloodpressure (cont); Diastolic bloodpressure (cont); Saturation (cont); Capillary refill (dich); Consciousness (normal / decreased / unconsciousness);
**Diagnostics, Therapy, Disposition**	Any lab (Yes/No); Any imaging (Yes/No); Any cultures (Yes/No); Oxygen therapy (Yes/No); Oral meds (Yes/No); Inhalation meds (Yes/No); IV meds or fluids (Yes/No); Immediate lifesaving interventions (Yes/No); Disposition (Mortality or ICU admission/ Admission / Other (cat)

Schinkelshoek G, *et al. BMJ Paediatrics Open* 2020; 4:e000687. doi: 10.1136/bmjpo-2020-000687

Supplementary figure 1. Missing data for the vital signs variables

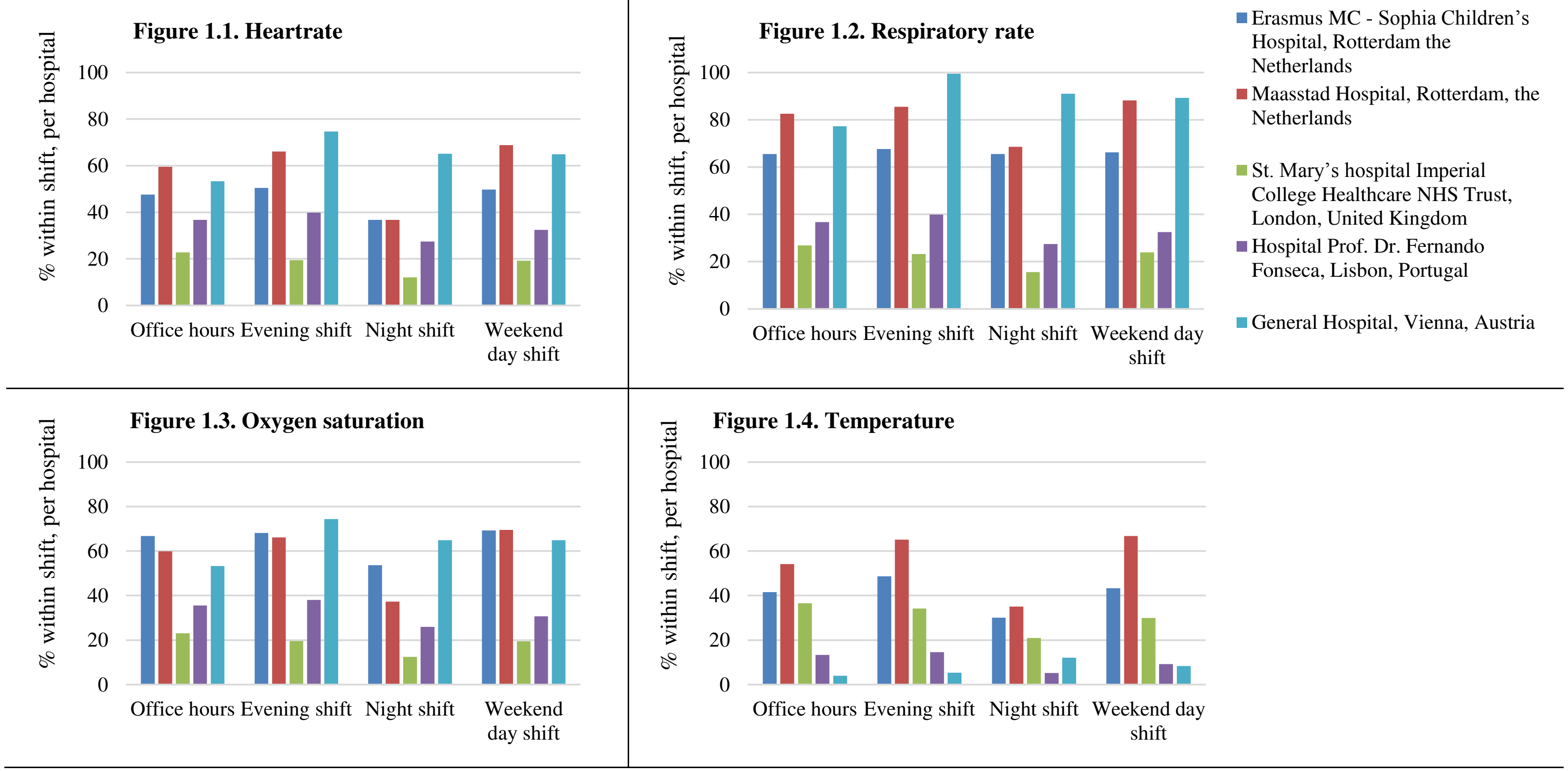

Schinkelshoek G, *et al. BMJ Paediatrics Open* 2020; 4:e000687. doi: 10.1136/bmjpo-2020-000687

Supplement 4. Complete case analyses

For the complete case analyses, we used a database consisting only of cases in which all vital signs (heart rate, respiratory rate, oxygen saturation and temperature) were measured and documented (total n=51,664). We performed the same regression analyses as with the imputation database, adjusted for age, gender, urgency, vital signs, presenting problem and hospital.

We found comparable results of the association between management and time of arrival at the ED, when comparing the results of the imputation dataset to the complete case dataset (Supplementary table 4). In the complete case analyses, imaging was less likely to be requested and treatment was more likely to be given during all out-of-office hours. During the night patients were significantly more likely to be admitted to the hospital. In complete case analyses, only the slightly lower number of laboratory diagnostics and slightly higher number of hospital admissions was not seen in weekend day shifts, but was confirmed in evening shifts. More chance variation in complete case analyses is likely to contribute to these differences. (6, 7)

Supplementary table 4 The association between management (lab, imaging, treatment, hospital and PICU[a] admission) and time of arrival at the ED, adjusted regression analyses. Complete cases (vital signs).

Total, n = 51,664<br>aOR[b] (95% CI)	**Office hours[c] (n=21,963)**	**Evening shift (n=7,800)**	**Night shift (n=14,251)**	**Day shift weekend (n=7,649)**
**Lab**	-<br>-	0.95<br>(0.89 – 1.00)	0.88<br>(0.81 – 0.95) *	1.02<br>(0.95 – 1.10)
**Imaging**	-<br>-	0.85<br>(0.80 – 0.91) *	0.63<br>(0.58 – 0.68) *	0.92<br>(0.85 – 0.99)*
**Treatment**	-<br>-	1.11<br>(1.05 – 1.17) *	1.46<br>(1.37 – 1.55) *	1.17<br>(1.10 – 1.24) *
**Hospital admission**	-<br>-	1.05<br>(0.97 – 1.14)	1.27<br>(1.16 – 1.39) *	0.95<br>(0.86 – 1.05)

[a] PICU = Paediatric Intensive Care Unit
[b]aOR = Adjusted Odds Ratio. Adjusted for age, gender, urgency, vital signs, presenting problem and hospital.
[c] Office hours (= day shift week) as reference shift.
* P-value ≤0.05. If not stated: p-value >0.05

Supplement 5. Hospital characteristics

Supplementary table 5.1 Patient characteristics - Erasmus MC - Sophia Children's Hospital, Rotterdam the Netherlands

(Total, n=18,590)	**Office hours[a] (n=8,915)**	**Evening shift (n=5,055)**	**Night shift (n=1,618)**	**Day shift weekend (n=3,002)**
**Median age, yrs *(IQR[b])***	4.6 (1.4 – 10.2)	4.1 (1.4 – 9.4)	3.7 (1.2 – 9.3)	4.2 (1.5 – 9.2)
**Gender, *n (%)***				
- **Male**	5,098 (57.2)	2,956 (58,5)	957 (59,1)	1,760 (58,6)
**MTS, *n (%)***				
- **High urgent**	1,073 (12.0)	655 (12.9)	356 (22.0)	343 (11.4)
- **Urgent**	4,040 (45.3)	2,502 (49.5)	741 (45.8)	1,461 (48.7)
- **Low urgent**	3,462 (38.8)	1,762 (34,8)	492 (30.4)	1,133 (37.7)
- **Missing**	340 (3.8)	136 (2.7)	29 (1.8)	65 (2.2)
**Vital signs, *n (%)***				
- **Normal**	2,925 (32.8)	1,559 (30.9)	440 (27.2)	962 (32.1)
- **1 Abnormal**	3,885(43.6)	2,114 (41.8)	681 (42.1)	1,289 (42.9)
- **2 or more abnormal**	2,105 (23.6)	1,381 (27.3)	497 (30.7)	751 (25.0)

[a]Office hours = Day shift week
[b]IQR = Interquartile range
[c]MTS = Manchester Triage System

Supplementary table 5.2 Patient characteristics - Maasstad Hospital, Rotterdam, the Netherlands

(Total, n=10,583)	**Office hours[a] (n=4,229)**	**Evening shift (n=3,630)**	**Night shift (n=981)**	**Day shift weekend (n=1,743)**
**Median age, yrs *(IQR[b])***	5.5 (1.8 – 11.4)	6.2 (2.2 – 11.7)	3.8 (1.0 – 10.5)	6.7 (2.5 – 11.9)
**Gender, *n (%)***				
- **Male**	2,384 (56.4)	1,999 (55.1)	573 (58.4)	1,048 (60.1)
**MTS, *n (%)***				
- **High urgent**	586 (13.9)	477 (13.2)	255 (26.0)	197 (11.3)
- **Urgent**	2,023 (47.8)	1,788 (49.3)	449 (45.8)	850 (48.8)
- **Low urgent**	1,567 (37.1)	1,339 (36.9)	267 (27.2)	684 (39.2)
- **Missing**	53 (1.3)	26 (0.7)	10 (1.0)	12 (0.7)
**Vital signs, *n (%)***				
- **Normal**	1,305 (30.9)	1,076 (29.7)	250 (25.5)	540 (31.0)
- **1 Abnormal**	1,839 (43.5)	1,526 (42.0)	387 (39.4)	747 (42.9)
- **2 or more abnormal**	1,085 (25.6)	1,028 (28.3)	344 (35.1)	456 (26.1)

[a]Office hours = Day shift week
[b]IQR = Interquartile range
[c]MTS = Manchester Triage System

Schinkelshoek G, *et al. BMJ Paediatrics Open* 2020; 4:e000687. doi: 10.1136/bmjpo-2020-000687

Supplementary table 5.3 Patient characteristics - St. Mary's hospital Imperial College Healthcare NHS Trust, London, United Kingdom

(Total, n=15,556)	**Office hours[a]** **(n=5,635)**	**Evening shift** **(n=4,847)**	**Night shift** **(n=2,429)**	**Day shift weekend** **(n=2,645)**
**Median age, yrs *(IQR[b])***	4.3 (1.5 – 9.6)	4.0 (1.5 – 8.9)	3.4 (1.3 – 7.4)	3.7 (1.5 – 7.7)
**Gender, *n (%)***				
- **Male**	3,137 (55.7)	2,736 (56.4)	1,328 (54.7)	1,476 (55.8)
**MTS, *n (%)***				
- **High urgent**	567 (10.1)	494 (10.2)	312 (12.8)	232 (8.7)
- **Urgent**	1,456 (25.8)	1,318 (27.2)	568 (23.4)	619 (23.4)
- **Low urgent**	3,612 (64.1)	3,035 (62.6)	1,549 (63.8)	1,794 (67.9)
- **Missing**	0 (0.0)	0 (0.0)	0 (0)	0 (0)
**Vital signs, *n (%)***				
- **Normal**	2,494 (44.3)	2,138 (44.1)	1,008 (41.5)	1,167 (44.1)
- **1 Abnormal**	2,152 (38.2)	1,801 (37.2)	887 (36.5)	1,010 (38.2)
- **2 or more abnormal**	989 (17.5)	909 (18.7)	534 (22.0)	468 (17.7)

[a]Office hours = Day shift week
[b]IQR = Interquartile range
[c]MTS = Manchester Triage System

Supplementary table 5.4 Patient characteristics - Hospital Prof. Dr. Fernando Fonseca, Lisbon, Portugal

(Total, n=53,175)	**Office hours[a]** **(n=22,470)**	**Evening shift** **(n=16,422)**	**Night shift** **(n=6,879)**	**Day shift weekend** **(n=7,404)**
**Median age, yrs *(IQR[b])***	5.2 (2.1 – 10.1)	4.7 (2.0 – 9.4)	3.8 (1.5 – 7.9)	4.3 (1.9 – 8.5)
**Gender, *n (%)***				
- **Male**	11,765 (52.4)	8,567 (52.2)	3,513 (51.1)	3,840 (51.9)
**MTS, *n (%)***				
- **High urgent**	2,310 (10.3)	1,935 (11.8)	1,191 (17.8)	756 (10.2)
- **Urgent**	4,281 (19.1)	3,844 (23.4)	1,445 (21.0)	1,381 (18.7)
- **Low urgent**	15,879 (70.6)	10,643 (64.8)	4,213 (61,3)	5,267 (71.1)
- **Missing**	0 (0.0)	0 (0.0)	0 (0.0)	0 (0.0)
**Vital signs, *n (%)***				
- **Normal**	8,740 (38.9)	5,924 (36.1)	2,374 (34.5)	2,700 (36.5)
- **1 Abnormal**	10,435 (46.4)	7,608 (46.3)	3,145 (45.7)	3,500 (47.3)
- **2 or more abnormal**	3,295 (14.7)	2,890 (17.6)	1,359 (19.8)	1,204 (16.2)

[a]Office hours = Day shift week
[b]IQR = Interquartile range
[c]MTS = Manchester Triage System

Schinkelshoek G, *et al. BMJ Paediatrics Open* 2020; 4:e000687. doi: 10.1136/bmjpo-2020-000687

**Supplementary table 5.5 Patient characteristics – General Hospital, Vienna, Austria**				
(Total, n=21,300)	**Office hours[a]** **(n=9,168)**	**Evening shift** **(n=6,475)**	**Night shift** **(n=2,231)**	**Day shift weekend** **(n=3,426)**
**Median age, yrs (*IQR[b]*)**	4.0 (1.6 – 8.5)	3.8 (1.5 – 8.4)	3.7 (1.4 – 8.3)	3.8 (1.7 – 7.6)
**Gender, *n (%)***				
- **Male**	4,766 (52.0)	3,440 (53.1)	1,195 (53.6)	1,832 (53.5)
**MTS, *n (%)***				
- **High urgent**	427 (4.6)	343 (5.3)	158 (7.1)	155 (4.5)
- **Urgent**	1,500 (16.4)	1,344 (20.8)	452 (20.3)	555 (16.2)
- **Low urgent**	6,958 (75.9)	4,297 (69.5)	1,371 (61.4)	2,488 (72.6)
- **Missing**	283 (3.1)	290 (4.5)	250 (11.2)	228 (6.7)
**Vital signs, *n (%)***				
- **Normal**	3,271 (35.7)	2,206 (34.1)	739 (33.1)	1,183 (34.5)
- **1 Abnormal**	4,233 (46.2)	2,899 (44.7)	1,000 (44.8)	1,573 (45.9)
- **2 or more abnormal**	1,664 (18.1)	1,371 (21.2)	493 (22.1)	670 (19.6)

[a]Office hours = Day shift week
[b]IQR = Interquartile range
[c]MTS = Manchester Triage System

Supplement 6. Stratified regression analyses

Supplementary table 6. Management adjusted regression analyses, per hospital					
Total n=119,204 aOR[a] (95% CI)	**Erasmus MC - Sophia Children's Hospital, Rotterdam the Netherlands** n = 18,590	**Maasstad Hospital, Rotterdam, the Netherlands** n = 10,583	**St. Mary's hospital Imperial College Healthcare NHS Trust, London, United Kingdom** n = 15,556	**Hospital Prof. Dr. Fernando Fonseca, Lisbon, Portugal** n = 53,175	**General Hospital, Vienna, Austria** n = 21,300
**Lab**					
- Evening shift	0.75 (0.69 – 0.82) *	0.82 (0.71 – 0.94) *	0.73 (0.63 – 0.83)*	1.00 (0.94 – 1.07)	0.61 (0.57 – 0.66) *
- Night shift	0.77 (0.68 – 0.87) *	0.86 (0.71 – 1.04)	0.71 (0.60 – 0.84) *	1.03 (0.95 – 1.11)	0.68 (0.61 – 0.77) *
- Day shift weekend	0.90 (0.81 – 0.99)*	0.85 (0.71 – 1.02)	0.68 (0.57 – 0.81)*	1.15 (1.07 – 1.25) *	0.82 (0.74 – 0.90) *
**Imaging**					
- Evening shift	0.73 (0.67 – 0.80) *	2.18 (1.93 – 2.47) *	0.77 (0.68 – 0.87) *	0.94 (0.88 – 0.99) *	0.59 (0.52 – 0.68) *
- Night shift	0.53 (0.46 – 0.62)*	1.05 0.84 – 1.30	0.52 (0.43 – 0.62) *	0.68 0.63 – 0.74 *	0.55 (0.44 – 0.67)*
- Day shift weekend	0.85 (0.76 – 0.94)	2.00 (1.72 – 2.34) *	0.81 (0.69 – 0.94) *	0.94 (0.88 – 1.02)	1.00 (0.86 – 1.16)
**Treatment**					
- Evening shift	1.13 (1.04 – 1.22) *	0.78 0.68 – 0.89) *	1.35 (1.25 – 1.47)*	0.99 (0.93 – 1.04)	1.44 (1.32 – 1.58) *
- Night shift	1.32 (1.17 – 1.50)*	1.03 (0.86 – 1.24)	1.55 (1.40 – 1.72) *	1.53 (1.43 – 1.64) *	2.32 (2.06 – 2.62) *
- Day shift weekend	1.12 1.01 – 1.23*	0.89 (0.75 – 1.06)	1.31 (1.19 – 1.54) *	1.12 (1.03 – 1.21) *	1.38 (1.23 – 1.55) *
**Hospital admission**					
- Evening shift	1.20 (1.09 – 1.31) *	1.05 (0.93 – 1.18)	0.92 (0.80 – 1.06)	1.05 (0.95 – 1.16)	0.93 (0.80 – 1.09)
- Night shift	1.43 (1.25 – 1.63) *	1.44 (1.22 – 1.71) *	0.95 (0.80 – 1.19)	1.51 (1.35 – 1.69) *	1.06 (0.86 – 1.30)
- Day shift weekend	1.15 (1.03 – 1.29 *	1.04 (0.89 – 1.21)	0.71 (0.59 – 0.85)*	1.09 (0.95 – 1.24)	1.32 (1.10 – 1.58) *

[a]aOR = Adjusted Odds Ratio. Adjusted for age, gender, urgency level, abnormal vital signs, presenting problem
*P-value ≤0.05. If not stated: p-value >0.05

1. Zachariasse JM, Seiger N, Rood PP, Alves CF, Freitas P, Smit FJ, et al. Validity of the Manchester Triage System in emergency care: A prospective observational study. PLoS One. 2017;12(2):e0170811.
2. Lee JY, Oh SH, Peck EH, Lee JM, Park KN, Kim SH, et al. The validity of the Canadian Triage and Acuity Scale in predicting resource utilization and the need for immediate life-saving interventions in elderly emergency department patients. Scand J Trauma Resusc Emerg Med. 2011 Nov 3;19:68.
3. van de Maat J, Jonkman H, van de Voort E, Mintegi S, Gervaix A, Bressan S, et al. Measuring vital signs in children with fever at the emergency department: an observational study on adherence to the NICE recommendations in Europe. Eur J Pediatr. 2020 Feb 8.
4. Gravel J, Opatrny L, Gouin S. High rate of missing vital signs data at triage in a paediatric emergency department. Paediatr Child Health. 2006 Apr;11(4):211-5.
5. Seiger N, van Veen M, Almeida H, Steyerberg EW, van Meurs AH, Carneiro R, et al. Improving the Manchester Triage System for pediatric emergency care: an international multicenter study. PLoS One. 2014;9(1):e83267.
6. Donders AR, van der Heijden GJ, Stijnen T, Moons KG. Review: a gentle introduction to imputation of missing values. J Clin Epidemiol. 2006 Oct;59(10):1087-91.
7. Sterne JA, White IR, Carlin JB, Spratt M, Royston P, Kenward MG, et al. Multiple imputation for missing data in epidemiological and clinical research: potential and pitfalls. Bmj. 2009 Jun 29;338:b2393.

Schinkelshoek G, *et al. BMJ Paediatrics Open* 2020; 4:e000687. doi: 10.1136/bmjpo-2020-000687
