# Supplementary material for: Management of children visiting the emergency department during out-of-office hours: an observational study
Source: BMJ Paediatr Open. 2020 Sep 15;4(1):e000687. doi: 10.1136/bmjpo-2020-000687 (PMC7493126; doi:10.1136/bmjpo-2020-000687)
Supplement: Author's manuscript [file bmjpo-2020-000687.draft_revisions.pdf]

BMJ Paediatrics Open

BMJ Paediatrics Open is committed to open peer review. As part of this commitment we make the peer review history of every article we publish publicly available.

When an article is published we post the peer reviewers' comments and the authors' responses online. We also post the versions of the paper that were used during peer review. These are the versions that the peer review comments apply to.

The versions of the paper that follow are the versions that were submitted during the peer review process. They are not the versions of record or the final published versions. They should not be cited or distributed as the published version of this manuscript.

BMJ Paediatrics Open is an open access journal and the full, final, typeset and author-corrected version of record of the manuscript is available on our site with no access controls, subscription charges or pay-per-view fees (http://bmjpaedsopen.bmj.com).

If you have any questions on BMJ Paediatrics Open's open peer review process please email info.bmjpo@bmj.com

BMJ Paediatrics Open

Management of children visiting the emergency department during out-of-office hours: an observational study

Journal:	*BMJ Paediatrics Open*
Manuscript ID	bmjpo-2020-000687
Article Type:	Original research
Date Submitted by the Author:	23-Mar-2020
Complete List of Authors:	Schinkelshoek, Gina; Erasmus MC – Sophia Children's Hospital<br>Borensztajn, Dorine; Erasmus MC – Sophia Children's Hospital, Department of Pediatrics<br>Zachariasse, Joany; Erasmus MC – Sophia Children's Hospital, Department of Pediatrics<br>Maconochie, Ian; Imperial College Hospital NHS Healthcare Trust, Paediatric Emergency Department<br>Alves, Claudio; Fernando Fonseca Hospital, General Paediatrics<br>Freitas , Paulo ; Hospital Fernando Fonseca , Intensive Care Unit<br>Smit, Frank; Maasstad hospital, Paediatrics<br>van der Lei, Johan; Erasmus MC, Department of Medical Informatics<br>Steyerberg, Ewout; Erasmus MC, Public Health<br>Greber-Platzer, Susanne; Medical University of Vienna, Division of Pediatric Pulmology, Allergology and Endocrinology, Department of Pediatrics and Adolescent Medicine<br>moll, Henriette; Erasmus MC – Sophia Children's Hospital, Department of Pediatrics
Keywords:	Accident & Emergency, General Paediatrics, Paediatric Practice

SCHOLARONE™
Manuscripts

I, the Submitting Author has the right to grant and does grant on behalf of all authors of the Work (as defined in the below author licence), an exclusive licence and/or a non-exclusive licence for contributions from authors who are: i) UK Crown employees; ii) where BMJ has agreed a CC-BY licence shall apply, and/or iii) in accordance with the terms applicable for US Federal Government officers or employees acting as part of their official duties; on a worldwide, perpetual, irrevocable, royalty-free basis to BMJ Publishing Group Ltd ("BMJ") its licensees and where the relevant Journal is co-owned by BMJ to the co-owners of the Journal, to publish the Work in this journal and any other BMJ products and to exploit all rights, as set out in our licence.

The Submitting Author accepts and understands that any supply made under these terms is made by BMJ to the Submitting Author unless you are acting as an employee on behalf of your employer or a postgraduate student of an affiliated institution which is paying any applicable article publishing charge ("APC") for Open Access articles. Where the Submitting Author wishes to make the Work available on an Open Access basis (and intends to pay the relevant APC), the terms of reuse of such Open Access shall be governed by a Creative Commons licence – details of these licences and which Creative Commons licence will apply to this Work are set out in our licence referred to above.

Other than as permitted in any relevant BMJ Author's Self Archiving Policies, I confirm this Work has not been accepted for publication elsewhere, is not being considered for publication elsewhere and does not duplicate material already published. I confirm all authors consent to publication of this Work and authorise the granting of this licence.

Confidential: For Review Only

MANAGEMENT OF CHILDREN VISITING THE EMERGENCY DEPARTMENT DURING OUT-OF-OFFICE HOURS: AN OBSERVATIONAL STUDY

Gina Schinkelshoek[1*], Dorine M. Borensztajn[1*], Joany M. Zachariasse[1], Ian K. Maconochie[2], Claudio F. Alves[3], Paulo Freitas[4], Frank J. Smit[5], Johan van der Lei[6], Ewout W. Steyerberg[7], Susanne Greber-Platzer[8], Henriëtte A. Moll[1]

Affliations:
[1]Erasmus MC – Sophia Children's Hospital, Department of General Paediatrics, Rotterdam, The Netherlands.
[2]Imperial College NHS Healthcare Trust, Department of Paediatric Accident and Emergency, London, United Kingdom.
[3]Hospital Prof. Dr. Fernando Fonseca, Department of Paediatrics, Lisbon, Portugal.
[4]Hospital Prof. Dr. Fernando Fonseca, Intensive Care Unit, Lisbon, Portugal.
[5]Maasstad Hospital, Department of Paediatrics, Rotterdam, the Netherlands.
[6]Erasmus MC- University Medical Centre Rotterdam, Department of Medical Informatics, Rotterdam, The Netherlands.
[7]Leiden University Medical Centre, Department of Biomedical Data Sciences, Leiden, The Netherlands.
[8]Medical University Vienna, Department of Paediatrics and Adolescent Medicine, Vienna, Austria.
*Gina Schinkelshoek and Dorine M. Borensztajn contributed equally to this paper.

Corresponding Author:
Prof. Dr. H.A. Moll
Department of General Paediatrics
Erasmus MC – Sophia Children's Hospital
P.O. Box 2060, 3000 CB, Rotterdam, The Netherlands.
Telephone: +31 10 703 61 96
Fax: +31 10 703 66 85
Email: H.A.Moll@erasmusmc.nl

Word count:
2515 words

Confidential: For Review Only

What is already known on this topic:

Concerns exist that emergency care may differ during out-of-office hours and office hours. There has been growing attention for paediatric emergency care at the emergency department recently. However, little is known about children visiting the emergency department during out-of-office hours.

What this study adds:

Children visiting the emergency department during the night were more seriously ill than during office hours. Fewer diagnostic tests, more treatment and more hospital admissions were reported during the night, which could not be explained by correcting for illness severity. These results are an important starting point for further research focusing on exploring the reasons for these differences and how these differences affect patient outcome.

Confidential: For Review Only

ABSTRACT

Background
The aim was to study the characteristics and management of children visiting the Emergency Department (ED) during out-of-office hours.

Methods
We analysed electronic health record data from 119.204 children visiting one of five EDs in four European countries. Patient characteristics and management (diagnostic tests, treatment, hospital admission and Paediatric Intensive Care Unit admission) were compared between children visiting during office hours and evening shifts, night shifts and weekend day shifts. Analyses were corrected for age, gender, MTS urgency, abnormal vital signs, presenting problem and hospital.

Results
Patients presenting at night were younger (median (IQR) age: 3.7 (1.4 – 8.2) years versus 4.8 (1.8 – 9.9)), more often classified as high urgent (15.8% versus 9.7%) and more often had ≥ 2 abnormal vital signs (22.8% versus 18.1%), compared to office hours. After correcting for disease severity, laboratory and radiological tests were less likely to be requested (aOR: 0.88, 95% CI: 0.83 – 0.92 and aOR: 0.65, 95% CI: 0.61 – 0.69 respectively); treatment was more likely to be undertaken (aOR: 1.66, 95% CI: 1.58 – 1.73) and patients were more likely to be admitted to the hospital (aOR: 1.37, 95% CI: 1.28 -1.46) at night. Patterns in management during out-of-office hours were comparable between the different hospitals.

Conclusions
Children visiting during the night are relatively more seriously ill, highlighting the need for optimal emergency care 24 hours a day. Further research is needed to explain the differences in management during the night and how these differences affect patient outcome.

Confidential: For Review Only

INTRODUCTION

Concerns exist that emergency care may differ during out-of-office hours, i.e. evening, night and/or weekend shifts, compared to office hours. For example, higher mortality rates were found during weekend visits in adult patients with sepsis presenting to the Emergency Department (ED).(1) Some suggest that worse patient outcome during out-of-office hours can, partly, be explained by higher illness severity and not by time of arrival as independent factor.(2, 3) When looking at the paediatric population, higher mortality rates were observed among children admitted to the PICU during out-of-office hours.(4) However, other studies analysing PICU patients are in conflict with this finding (5-9).

Over the past few years, increasing attention has been paid to paediatric emergency care, leading to different guidelines and standards for children visiting the ED.(10-14) These guidelines aim to achieve optimal utilization of available resources, optimize patient outcome and thereby improve quality of care. A recent study reported that main standards of care are fulfilled in European EDs, but some areas of improvement still exist.

However, little research has focused specifically on children visiting the ED during out-of-office hours. Recently, variation in paediatric ED utilization patterns and the presentation of these children by shift of arrival has been reported.(15) But this study was a single centre study, which may limit the generalizability. Moreover, no study has analysed resource utilization at paediatric EDs during out-of-office hours, except for studies analysing children with a specific presenting problem.(16-18)

It is important to obtain insight into the characteristics and management of the whole paediatric patient population attending the ED with its variable presenting problems, as this may be helpful in optimizing guidelines and resource allocation, and thereby in improving the quality of care on a 24 hours a day basis.

Confidential: For Review Only

The aim of this study was to compare patient characteristics and management of paediatric patients visiting the ED during out-of-office hours to office hours, in four different European countries.

Confidential: For Review Only

METHODS

Study design, setting, patients

This study is part of the TrIAGE project (Triage Improvement Across General Emergency departments for paediatric patients), a prospective observational study. This project captured routinely collected, standardized, electronic health record data of all non-scheduled ED visits of children <16 years in five different hospitals in four different countries. The five participating hospitals were: Erasmus Medical Centre, the Netherlands; Maasstad Hospital, the Netherlands; St. Mary's hospital Imperial College Healthcare NHS Trust, United Kingdom; Hospital Prof. Dr. Fernando Fonseca, Portugal; Vienna General Hospital, Austria. The enrolment period varied from 8 to 36 months between 2012 and 2015.

At the beginning of the study, a minimum dataset of variables for collection was defined. If necessary, entries in the electronic health data were added or modified. Nurses at the ED were informed about the study and encouraged to fill in the entire medical records, including vital signs, which is in principle routine data. During the study this routinely collected data was documented in the electronic, medical records by nurses at the ED. Data completeness and accuracy of the data was reviewed using a checklist of quality control. Sometimes, data was completed by information or vital signs from medical records documented by physicians at the ED. Coded data were extracted from the electronic hospital information systems and thereafter transferred to the principal investigator as an encrypted file. Careful data harmonization and quality checks were conducted. The requirement for informed consent was obtained by all hospitals and the study was approved by the medical ethical committees of all participating institutions.

Patient and Public Involvement statement

Patients or the public were not involved in this research.

Confidential: For Review Only

Definitions

Office hours versus out-of-office hours

Office hours were defined as Monday until Friday, between 08:00 am and 05:59 pm, also referred to as day shift week. We compared office hours, as reference shift, to out-of-office hours, divided into evening shift, night shifts and day shifts during the weekend. Evening shifts were defined as Monday until Sunday between 6:00 pm and 10:59 pm and night shifts as Monday until Sunday between 11:00 pm and 07:59 am, based on previous literature. (5, 9, 16) Weekend day shifts were defined as Saturday and Sunday between 08:00 am and 5:59 pm.

Patient characteristics

For this study, the following data were extracted from the database: age, gender, time and date of arrival, Manchester Triage System (MTS) urgency, vital signs and presenting symptom (based on the MTS flowchart). The MTS urgency consists of five categories: emergent, very urgent, urgent, standard and non-urgent.(19-21) These categories were divided in three groups: high urgent (emergent or very urgent, <10 minutes waiting time), urgent (urgent, <60 minutes waiting time), low urgent (standard or non-urgent, ≥ 60 minutes waiting time) . Vital signs included heart rate, respiratory rate, oxygen saturation and temperature. Vital signs were considered abnormal based on the Advanced Paediatric Life Support (APLS) reference values, with fever defined as temperature ≥38.5ºC.(22) The MTS flowchart was used to create subgroups of presenting problems. These subgroups, based on a previous study (23), included medical (dyspnoea, ear, nose and throat (ENT) problems, gastro-intestinal problems, neurological problems, unwell child, urinary tract problems, rash, abscess/infection, others) and trauma (trauma and wounds).

Confidential: For Review Only

Management

Management was defined as diagnostics, (laboratory tests and imaging), treatment at the ED and hospital admission directly after ED visit. Laboratory tests were divided into: blood tests, including cultures; and urine tests, including cultures. Imaging was divided into: x-ray, ultrasound and Computed Tomography (CT).

Treatment was divided into: oral medication, inhalation medication, intravenous (IV) medication or IV fluids and Immediate Lifesaving Interventions (ILI) (see supplementary table 1 (24)).

In the multivariable regression models, we defined laboratory diagnostics as any form of laboratory tests, radiological diagnostics as any form of imaging and treatment as any form of treatment.

Hospital admission included patients who were admitted to the general ward or to the PICU. PICU admission was defined as admission to the PICU directly from the ED.

Statistical analysis

In descriptive statistics of patient characteristics continuous, skewed variables are shown as median with interquartile range (IQR). Nominal and ordinal variables are shown as number of patients with percentage within shift. Nominal and ordinal variables considering management are shown as percentages within shift, with inter-hospital ranges.

In multivariable regression analyses differences in management were compared between office hours and evening, night and weekend day shifts. To study the independent role of shift of arrival, these analyses were adjusted for age, gender, MTS urgency, abnormal vital signs, flowchart (trauma or medical) and hospital. We performed multivariable regression analyses for each hospital separately and multivariable regression analyses with an interaction term of

Confidential: For Review Only

hospital and shift, to study inter-hospital differences. Missing vital signs were imputed using a multiple imputation model. (supplementary table 2 (25))

Results were considered statistically significant at a P-value of ≤0.05.

Initial analyses were performed with SPSS software, version 21 (IBM SPSS statistics, IBM corporation, Armonk, NY).

Confidential: For Review Only

RESULTS

Patient population

In total, 119.209 patients, ≤16 years, visited the participating EDs. Five patients were excluded owing to missing arrival time or date, leaving 119.204 patients for analysis, with 50.417 (42.3%) of these patients who visited the ED during office hours versus 68.787 (57.7%) during out-of-office hours. Of those, 18.220 (15.3%) visited during weekend day shifts, 36.429 (30.6%) during the evening and 14.138 (11.9%) during the night.

Patients who visited the ED during the night were younger, were more often classified by MTS as high urgent, and more often had two or more abnormal vital signs (table 1).

During office hours, unwell child (19.8%), trauma (18.1%) and gastro-intestinal problems (15.1%) were the most common presenting problems (figure 1). During the night, relatively more unwell child (24.3%), gastro-intestinal problems (20.2%), dyspnoea (17.6%) and fewer trauma cases (7.6%) were observed. Presenting problems during the evening and weekend day shifts were similar compared to office hours. (supplementary table 3)

Management

During out-of-office hours, fewer diagnostic tests, more treatment and more hospital and PICU admissions were conducted. (table 2)

During the evening and weekend day shift, fewer urine and blood tests were performed.

During the evening, night and weekend day shifts, imaging was performed less often, except for X-ray during the evening shift. Treatment with oral, inhaled and intravenous medication were given more often, especially during the night. Also, during the night, slightly more immediate lifesaving interventions were seen, whilst more hospital and ICU admissions were observed.

Confidential: For Review Only

In multivariable regression analyses, the results were adjusted for age, gender, urgency, vital signs and presenting problem (medical or trauma) at triage and hospital. (table 3) Laboratory and imaging diagnostics were significantly less likely to be performed during the evening, night and weekend day shifts. Also, patients were significantly more likely to receive treatment during all out-of-office hours shifts and more likely to be admitted to the hospital during the evening and night shift. After adjusting, no association was found between PICU admission and out-of-office hours visits.

Inter-hospital analyses

Hospital characteristics are shown in table 4. Some inter-hospital variation in management was observed. (table 2) When adjusting for age, gender, urgency, vital signs and presenting problem (medical or trauma) at triage, overall patterns in management during out-of-office hours were comparable between the different hospitals, with some variation remaining. (supplementary table 4) We found a significant interaction effect of hospital and shift in the adjusted regression analyses with laboratory diagnostics, imaging diagnostics, treatment and hospital admission, but not with PICU admission. (results not shown)

Confidential: For Review Only

DISCUSSION

In this large observational study of 119.204 visits in five EDs in Europe, we demonstrate differences in illness severity and management of children visiting the ED during out-of-office hours compared to office hours.

We found that children visiting during the night were more seriously ill. Secondly, after correcting for patient characteristics, illness severity and hospital, we observed fewer laboratory and radiological tests whilst children were more likely to receive treatment at the ED and to be admitted to the hospital during especially the night. No association was found between out-of-office hours visits and PICU admission, after statistically correcting for disease severity. Overall, the trends in out-of-office hours care were comparable between the different hospitals. In a recent study, ED utilization patterns of paediatric patients through the day was analysed.(15) The distribution of visits over the evening and night shifts was quite similar to our results. Also, in line with our results, they observed that children during the night are relatively more ill. So, although the proportion of patients visiting during the night is the smallest, the acuity of this group is the highest, which shows the importance of the same quality of care on a 24 hours a day basis. In contrast to our results, no association between shift of arrival and hospital admission was reported. However, this was a single centre study, which limits the generalizability.

Variation in management during out-of-office hours, most pronounced during the night, could not be explained by illness severity. Reducing variation in care could result in lower resource utilization and costs, and could improve the use of time, space and staff. (26) To obtain more standardized care, also during out-of-office hours, it is essential to understand which factors contribute to the differences we observed.

At first, different levels of staff during the night might influence management. Several studies found differences in resource use depending on physician background and experience.(27-31)

https://mc.manuscriptcentral.com/bmjpo

Confidential: For Review Only

We did stratify our regression analyses for hospital, thereby allowing for inter-hospital variations in, for example, staff level. However, we did not have the specific information about experience and staff level for each individual visit at the different hospitals. Secondly, availability of resources during night shifts, such as imaging resources or the availability of specialized radiologists to assess these results during the night, might contribute to the lower use of imaging during the night. (32)

Our results raise the question whether these higher treatment and admission rates, and the lower use of diagnostic imaging during the night, might have been unwarranted and contribute to unnecessary costs. Earlier research reported that higher resource utilization is related to higher costs, but not to lower admission rates. (33-35) One of the hospitals in our study showed no difference in diagnostics during the night, in contrast to the other hospitals, but still showed higher admission rates compared to office hours.

We speculate that our results might also partly be explained by a more pragmatic approach of the patient during the night, resulting in fewer diagnostic tests, more treatment and more hospital admissions. Previous literature did report higher rates of antibiotic prescriptions (16) and recommendations to admit to the hospital (36), in children visiting the ED during the night. Additionally, parental demand might contribute to these results. (37) Furthermore, in contrast to general hospital admission, the higher rates of PICU admission during the night, could be explained by illness severity and case mix. The indications for PICU admissions are more standardized compared to indications for general hospital and therefore less influenced by non-patient factors.

These results point to the need for further research to explain the differences in clinical decisions during the night, by assessing differences in standards of care during out-of-office hours, such as availability of experienced staff and availability of diagnostics and equipment.(14) Moreover, it is important to obtain insight in how these differences in

Confidential: For Review Only

management affect patient outcome, for example rehospitalisation, morbidity and 30-day mortality. Implementing guidelines for management of children with specific conditions, could help to reduce variation in management within and between hospitals, which optimize resource allocation and costs. Moreover, these guidelines could allow for better comparisons between the hospitals in further research.

Other studies on the topic of out-of-office hours care, including adult patients at the ED and paediatric patients at the PICU, described worse patient outcome during the night and evening, represented by higher mortality rates. (1, 4, 38) We did not use mortality as the main outcome as the mortality in our dataset was very low (16 patients, 0.01%), which is in line with previous literature. (39, 40)

Strengths and limitations

A major strength of this study is the large number of patients from different centres, including both university as teaching hospitals. This increases the generalizability of the results. Moreover, we adjusted for multiple variables concerning illness severity to analyse the independent role of time of visit to management.

To appreciate the results, some limitations have to be considered. The analyses were based on routinely collected data, resulting in some missing data, which we dealt with using multiple imputation. Also, there are possibly more variables representing illness severity and influencing management, that we did not include, for example comorbidity, bed availability or (experience level of) staff.

Conclusion

Children visiting during the night are relatively more seriously ill than those visiting during office hours. This highlights the need for optimal emergency care on a 24 hours a day basis.

Confidential: For Review Only

Furthermore, differences in management are present depending on the time of ED visit. Especially during the night, diagnostic tests are performed less frequently, whilst treatment is initiated more often and more children are admitted to the hospital than during office hours. These patterns are not explained by illness severity. Further research should focus on exploring the reasons for these differences and how these differences affect patient outcome.

Confidential: For Review Only

FUNDING STATEMENT

This research received no specific grant from any funding agency in the public, commercial or not-for-profit sectors.

COMPETING INTERESTS

All authors have no conflicts of interest relevant to this article to disclose.

CONTRIBUTORSHIP STATEMENT

Gina Schinkelshoek: Conceptualization, data curation, formal analysis, investigation, writing original draft, review and editing.

Dorine M. Borensztajn: Conceptualization, data curation, formal analysis, investigation, writing original draft, review and editing.

Joany M. Zachariasse: Data curation, formal analysis, investigation, review and editing.

Susanne Greber-Platzer: Data curation, investigation, review and editing.

Claudio F. Alves: Data curation, investigation, review and editing.

Paulo Freitas: Data curation, investigation, review and editing.

Frank J. Smit: Data curation, investigation, review and editing.

Ian K. Maconochie: Data curation, investigation, review and editing.

Johan van der Lei: Conceptualization, data curation, review and editing.

Ewout W. Steyerberg: Data curation, review and editing.

Henriëtte A. Moll: Conceptualization, data curation, investigation, project administration, supervision, review and editing.

All authors approved the final manuscript as submitted and agree to be accountable for all aspects of work.

Confidential: For Review Only

DATA SHARING STATEMENT

Data from this study are available upon request to the corresponding author of the study (h.a.moll@erasmusmc.nl), subject to local rules and regulations.

Confidential: For Review Only

References

1. Powell ES, Khare RK, Courtney DM, Feinglass J. The weekend effect for patients with sepsis presenting to the emergency department. J Emerg Med. 2013 Nov;45(5):641-8.
2. Mikulich O, Callaly E, Bennett K, O'Riordan D, Silke B. The increased mortality associated with a weekend emergency admission is due to increased illness severity and altered case-mix. Acute Med. 2011;10(4):182-7.
3. Walker AS, Mason A, Quan TP, Fawcett NJ, Watkinson P, Llewelyn M, et al. Mortality risks associated with emergency admissions during weekends and public holidays: an analysis of electronic health records. Lancet. 2017 May 09.
4. Arias Y, Taylor DS, Marcin JP. Association between evening admissions and higher mortality rates in the pediatric intensive care unit. Pediatrics. 2004 Jun;113(6):e530-4.
5. Peeters B, Jansen NJ, Bollen CW, van Vught AJ, van der Heide D, Albers MJ. Off-hours admission and mortality in two pediatric intensive care units without 24-h in-house senior staff attendance. Intensive Care Med. 2010 Nov;36(11):1923-7.
6. McShane P, Draper ES, McKinney PA, McFadzean J, Parslow RC, Paediatric Intensive Care Audit N. Effects of out-of-hours and winter admissions and number of patients per unit on mortality in pediatric intensive care. J Pediatr. 2013 Oct;163(4):1039-44 e5.
7. Thabet FC, alHaffaf FA, Bougmiza IM, Bafaqih HA, Chehab MS, alMohaimeed SA. Off-Hours Admissions and Mortality in PICU Without 24-Hour Onsite Intensivist Coverage. J Intensive Care Med. 2018 Jan 1:885066618778824.
8. Hixson ED, Davis S, Morris S, Harrison AM. Do weekends or evenings matter in a pediatric intensive care unit? Pediatr Crit Care Med. 2005 Sep;6(5):523-30.
9. Numa A, Williams G, Awad J, Duffy B. After-hours admissions are not associated with increased risk-adjusted mortality in pediatric intensive care. Intensive Care Med. 2008 Jan;34(1):148-51.
10. American Academy of Pediatrics Committee on Pediatric Emergency M, American College of Emergency Physicians Pediatric C, Emergency Nurses Association Pediatric C. Joint policy statement--guidelines for care of children in the emergency department. Ann Emerg Med. 2009 Oct;54(4):543-52.
11. Gausche-Hill M, Wiebe RA. Guidelines for preparedness of emergency departments that care for children: A call to action. Annals of Emergency Medicine. [doi: 10.1067/mem.2001.114069]. 2001;37(4):389-91.
12. Remick K, Gausche-Hill M, Joseph MM, Brown K, Snow SK, Wright JL, et al. Pediatric Readiness in the Emergency Department. Pediatrics. 2018 Nov;142(5).
13. Facing the Future: Standards for children in emergency care settings. Royal College of Paediatrics and Child Health; 2018 [cited 2019 October]; Available from: https://www.rcpch.ac.uk/sites/default/files/2018-06/FTFEC%20Digital%20updated%20final.pdf.
14. Standards of Care for Children in Emergency Departments (Third version). International Federation for Emergency Medicine; 2012 [updated April 2019; cited 2019 October]; Available from: https://www.ifem.cc/wp-content/uploads/2019/06/Standards-of-Care-for-Children-in-Emergency-Departments-V3-2019.pdf.
15. Almubarak H, Meckler G, Doan Q. Factors and outcomes associated with paediatric emergency department arrival patterns through the day. Paediatr Child Health. 2019 Aug;24(5):323-9.
16. Roggen I, van Berlaer G, Gordts F, Pierard D, Hubloue I. Acute sore throat in children at the emergency department: best medical practice? Eur J Emerg Med. 2015 Oct;22(5):343-7.

Confidential: For Review Only

17. Johnson EK, Graham DA, Chow JS, Nelson CP. Nationwide emergency department imaging practices for pediatric urolithiasis: room for improvement. J Urol. 2014 Jul;192(1):200-6.
18. Daverio M, Bressan S, Gregori D, Babl FE, Mackay MT. Patient and Process Factors Associated With Type of First Neuroimaging and Delayed Diagnosis in Childhood Arterial Ischemic Stroke. Acad Emerg Med. 2016 Sep;23(9):1040-7.
19. De Caluwé R, Manchester Triage G. Triage voor de spoedeisende hulp: Elsevier Gezondheidszorg; 2007.
20. van Veen M, Steyerberg EW, van't Klooster M, Ruige M, van Meurs AHJ, van der Lei J, et al. The Manchester triage system: improvements for paediatric emergency care. Emergency Medicine Journal. 2012;29(8):654-9.
21. Mackway-Jones K, Marsden J, Windle J. Emergency Triage: Manchester Triage Group. Third ed. Oxford, United Kingdom: John Wiley & Sons; 2013.
22. Turner NM. Advanced paediatric life support : de Nederlandse editie. Vijfde druk. ed. Houten :: Bohn Stafleu van Loghum; 2017.
23. Seiger N, van Veen M, Almeida H, Steyerberg EW, van Meurs AH, Carneiro R, et al. Improving the Manchester Triage System for pediatric emergency care: an international multicenter study. PLoS One. 2014;9(1):e83267.
24. Lee JY, Oh SH, Peck EH, Lee JM, Park KN, Kim SH, et al. The validity of the Canadian Triage and Acuity Scale in predicting resource utilization and the need for immediate life-saving interventions in elderly emergency department patients. Scand J Trauma Resusc Emerg Med. 2011 Nov 3;19:68.
25. Donders AR, van der Heijden GJ, Stijnen T, Moons KG. Review: a gentle introduction to imputation of missing values. J Clin Epidemiol. 2006 Oct;59(10):1087-91.
26. Neuman MI, Chiang VW. Variation in pediatric care at US hospitals. Pediatrics. 2013 Aug;132(2):369-70.
27. Pitts SR, Morgan SR, Schrager JD, Berger TJ. Emergency department resource use by supervised residents vs attending physicians alone. Jama. 2014 Dec 10;312(22):2394-400.
28. White AL, Armstrong PA, Thakore S. Impact of senior clinical review on patient disposition from the emergency department. Emerg Med J. 2010 Apr;27(4):262-5, 96.
29. Hampers LC, Faries SG. Practice variation in the emergency management of croup. Pediatrics. 2002 Mar;109(3):505-8.
30. Melo MR, Ferreira-Magalhaes M, Flor-Lima F, Rodrigues M, Severo M, Almeida-Santos L, et al. Dedicated Pediatricians in Emergency Department: Shorter Waiting Times and Lower Costs. PLoS One. 2016;11(8):e0161149.
31. Knapp JF, Simon SD, Sharma V. Variation and trends in ED use of radiographs for asthma, bronchiolitis, and croup in children. Pediatrics. 2013 Aug;132(2):245-52.
32. Fullerton K, Depinet H, Iyer S, Hall M, Herr S, Morton I, et al. Association of Hospital Resources and Imaging Choice for Appendicitis in Pediatric Emergency Departments. Acad Emerg Med. 2017 Apr;24(4):400-9.
33. Kharbanda AB, Hall M, Shah SS, Freedman SB, Mistry RD, Macias CG, et al. Variation in resource utilization across a national sample of pediatric emergency departments. J Pediatr. 2013 Jul;163(1):230-6.
34. Florin TA, French B, Zorc JJ, Alpern ER, Shah SS. Variation in emergency department diagnostic testing and disposition outcomes in pneumonia. Pediatrics. 2013 Aug;132(2):237-44.
35. Bourgeois FT, Monuteaux MC, Stack AM, Neuman MI. Variation in emergency department admission rates in US children's hospitals. Pediatrics. 2014 Sep;134(3):539-45.

Confidential: For Review Only

36. El Khoury AC, Durden E, Ma L, Markson LE, Lee AW, Duan Y, et al. Perception and management of fever in infants up to six months of age: a survey of US pediatricans. BMC Pediatr. 2010 Dec 22;10:95.
37. Pachter LM, Ludwig S, Groves A. Night people: utilization of a pediatric emergency department during the late night. Pediatr Emerg Care. 1991 Feb;7(1):12-4.
38. Duvald I, Moellekaer A, Boysen MA, Vest-Hansen B. Linking the severity of illness and the weekend effect: a cohort study examining emergency department visits. Scand J Trauma Resusc Emerg Med. 2018 Sep 5;26(1):72.
39. Lopez E, Udaondo J, Olabarri M, Martinez-Indart L, Benito J, Mintegi S, et al. Mortality in Spanish pediatric emergency departments: a 5-year multicenter survey. Eur J Emerg Med. 2017 Dec;24(6):392-7.
40. O'Malley P, Barata I, Snow S, American Academy of Pediatrics Committee on Pediatric Emergency M, American College of Emergency Physicians Pediatric Emergency Medicine C, Emergency Nurses Association Pediatric C. Death of a child in the emergency department. Pediatrics. 2014 Jul;134(1):e313-30.

Confidential: For Review Only

Table 1. Patient characteristics				
(Total, n=119.204)	**Office hours[a] (n=50.417)**	**Evening shift (n=36.429)**	**Night shift (n=14.138)**	**Day shift weekend (n=18.220)**
**Median age, yrs *(IQR[b])***	4.8 (1.8 – 9.9)	4.5 (1.8 – 9.5)	3.7 (1.4 – 8.2)	4.2 (1.7 – 8.8)
**Gender, *n (%)***				
- **Male**	27.150 (53.9)	19.698 (54.1)	7.566 (53.5)	9.956 (54.6)
**MTS, *n (%)***				
- **High urgent**	4.870 (9.7)	3.802 (10.4)	2236 (15.8)	1.636 (9.0)
- **Urgent**	13.393 (26.6)	10.899 (29.9)	3.721 (26.3)	4.913 (27.0)
- **Low urgent**	31.478 (61.7)	21.276 (58.4)	7.892 (55.8)	11.366 (62.4)
- **Missing**	676 (1.3)	452 (1.2)	289 (2.0)	305 (1.7)
**Vital signs, *n (%)[d]***				
- **Normal**	18.734 (37.2)	12.903 (35.4)	4.812 (34.0)	6.553 (36.0)
- **1 Abnormal**	22.545 (44.7)	15.947 (43.8)	6.100 (43.1)	8.119 (44.6)
- **2 or more abnormal**	9.139 (18.1)	7.578 (20.8)	3.227 (22.8)	3.548 (19.5)

[a]Office hours = Day shift week
[b]IQR = Interquartile range
[c]MTS = Manchester Triage System
[d]Missing vital signs were imputed using a multiple imputation model including age, vital sign values (heart rate 42.7%, respiratory rate 52.4%, oxygen saturation 44.9% and temperature 22.5% missing values), MTS urgency, presenting problem and disposition after the ED.

For Peer Review Only

**Table 2. Management (diagnostics, treatment, hospital admission and PICU admission), descriptive**				
Total, n = 119.204	**Office hours[a] (n=50.417)**	**Evening shift (n=36.429)**	**Night shift (n=14.138)**	**Day shift weekend (n=18.220)**
**Mean % (inter-hospital range)**				
**Laboratory diagnostics**				
**Any lab test (n=119.204)**	22.2 (11.7 – 40.6)	18.6 (9.2 – 33.0)	21.5 (9.8 – 40.0	20.2 (8.0 – 33.3)
**Urine tests (n=50.473)[b]**	20.2 (11.0 - 23.5)	17.1 (8.1 – 22.3)	20.2 (13.9 – 23.2)	16.6 (7.7 – 19.6)
**Blood tests (n=66.029)[b]**	21.9 (11.7 – 26.7)	15.2 (9.2 – 20.3)	20.4 (9.8 - 30.5)	17.2 (7.9 – 23.5)
**Imaging diagnostics**				
**X-ray (n=119.204)**	19.5 (6.0 - 24.7)	19.4 (3.7 – 40.9)	12.1 (4.3 – 17.1)	17.4 (5.5 -41.2)
**Ultrasound (n=119.204)**	2.7 (1.5 - 6.0)	1.7 (0.5 - 2.9)	1.2 (0.5 - 4.1)	2.2 (0.6 - 4.2)
**CT (n=119.204)**	1.2 (0.7 - 2.5)	1.0 (0.6 - 2.4)	0.9 (0.4 - 2.3)	1.0 (0.5 - 2.1)
**Treatment**				
**Oral medication (n=108.621)[b]**	13.9 (8.0 – 34.3)	17.2 (8.6 – 41.8)	23.5 (14.9 – 45.1)	16.5 (8.2 – 39.7)
**Inhaled medication (n=119.204)**	6.5 (3.6 - 8.7)	6.5 (3.5 – 9.0)	12.0 (6.0 – 15.4)	6.9 (3.5 – 9.8)
**IV[c] medication or fluids (n=119.204)**	7.3 (4.1 - 14.0)	7.0 (4.2 - 11.3)	11.3 (4.6 - 19.4)	7.8 (3.4 - 12.0)
**ILI[d] (n=119.204)**	0.3 (0.0 – 1.1)	0.3 (0.0 -1.2)	0.5 (0.0 -1.9)	0.4 (0.1 -1.2)
**Hospital admission (n=119.204)**				
**Yes**	9.7 (4.3 - 22.6)	10.2 (5.0 – 24.2)	14.3 (8.8 – 38.4)	9.9 (4.6 – 22.4)
**PICU admission (n=119.204)**				
**Yes**	0.6 (0.1 – 2.3)	0.5 (0.1 -2.8)	0.8 (0.2 - 4.7)	0.6 (0.0 - 2.7)

[a]Office hours = Day shift week
[b]Not all variables were complete for all hospitals, making the total number less than 119.204.
[c] IV = Intravenous
[d] ILI = Immediate Lifesaving Interventions

Confidential: For Review Only

Table 3. The association between management (lab, imaging, treatment, hospital and PICU[a] admission) and time of arrival at the ED, adjusted regression analyses

Total, n = 119.204 aOR[b] (95% CI)	**Office hours[c] (n=50.417)**	**Evening shift (n=36.429)**	**Night shift (n=14.138)**	**Day shift weekend (n=18.220)**
**Lab**	- -	0.81 (0.78 – 0.84) *	0.88 (0.83 – 0.92) *	0.88 (0.84 – 0.93) *
**Imaging**	- -	0.87 (0.83 – 0.90) *	0.65 (0.61 – 0.69) *	0.91 (0.87 – 0.95) *
**Treatment**	- -	1.09 (1.05 – 1.12) *	1.66 (1.58 – 1.73) *	1.13 (1.09 – 1.18) *
**Hospital admission**	- -	1.06 (1.01 – 1.11) *	1.36 (1.28 – 1.45) *	1.03 (0.97 – 1.10)
**PICU[a] admission**	- -	1.16 (0.95 – 1.41)	0.97 (0.76 – 1.26)	1.17 (0.91 – 1.49)

[a] PICU = Paediatric Intensive Care Unit
[b]aOR = Adjusted Odds Ratio. Adjusted for age, gender, urgency, vital signs, flowchart (medical/trauma) and hospital. Missing vital signs were imputed using a multiple imputation model including age, vital sign values (heart rate 42.7%, respiratory rate 52.4%, oxygen saturation 44.9% and temperature 22.5% missing values), MTS urgency, presenting problem and disposition after the ED.
[c] Office hours (= day shift week) as reference shift.
* P-value ≤0.05. If not stated: p-value >0.05

Confidential: For Review Only

Table 4. Hospital characteristics

	Erasmus MC - Sophia Children's Hospital, Rotterdam the Netherlands	Maasstad Hospital, Rotterdam, the Netherlands	St. Mary's hospital Imperial College Healthcare NHS Trust, London, United Kingdom	Hospital Prof. Dr. Fernando Fonseca, Lisbon, Portugal	General Hospital, Vienna, Austria
**Hospital characteristics**	University hospital 60 paediatric beds	Teaching hospital 59 paediatric beds	University hospital 46 paediatric beds	Community hospital 91 paediatric beds	University hospital 134 paediatric beds
**Emergency department characteristics[a]**	6.500 children/year	9.500 children/year	27.000 children/year	60.000 children/year	22.000 children/year
**Number of patients included**	18.590	10.583	15.556	53.175	21.300
**Inclusion period**	01-01-2012 to 31-12-2014	01-05-2014 to 31-10-2015	01-07-2014 to 28-02-2015	01-03-2014 to 28-02-2015	01-01-2014 to 31-12-2014
**Number of patients during:**					
**- Office hours**	8.915 (48.0%)	4.229 (40.0%)	5.635 (36.2%)	22.470 (42.3%)	9.168 (43.0%)
**- Out-of-office hours**	9.675 (52.0%)	6.354 (60.0%)	9.921 (63.8%)	30.705 (57.7%)	12.132 (57.0%)
**Median age, *yrs (IQR[b])***	4.3 (1.4 – 9.8)	5.7 (1.9 – 11.6)	3.9 (1.5 – 8.8)	4.7 (1.9 – 9.5)	3.9 (1.6 – 8.3)
**MTS, *n (%)***					
- **High urgent**	2.249 (12.1)	1.498 (14.1)	1.571 (10.1)	6.143 (11.5)	1.083 (5.0)
- **Urgent**	8.922 (48.0)	5.127 (48.4)	3.995 (25.7)	11.030 (20.7)	3.852 (18.1)
- **Low urgent**	6.849 (36.8)	3.857 (36.4)	9.990 (64.2)	36.002 (67.7)	15.314 (71.9)
- **Missing**	570 (3.1)	101 (0.1)	0 (0.0)	0 (0.0)	1.051 (4.9)
**Presenting problem, *n (%)***					
- **Medical**	13.547 (73.0)	5.923 (56.0)	12.231 (78.6)	45.917 (86.4)	19.705 (92.6)
- **Trauma**	4.473 (24.1)	4.561 (43.1)	3.325 (21.4)	7.258 (13.6)	503 (2.4)
- **Missing**	550 (3.0)	99 (0.9)	0 (0.0)	0 (0.0)	1.082 (5.1)

[a]All emergency departments (ED) are paediatric only, except for the Maasstad Hospital, which is mixed adult-paediatric. The Erasmus MC-Sophia Children's hospital has a mixed adult-paediatric ED since October 2014 (3 months in total during the inclusion period).
[b]IQR = Interquartile range.

Confidential: For Review Only

Figure 1. Presenting problems

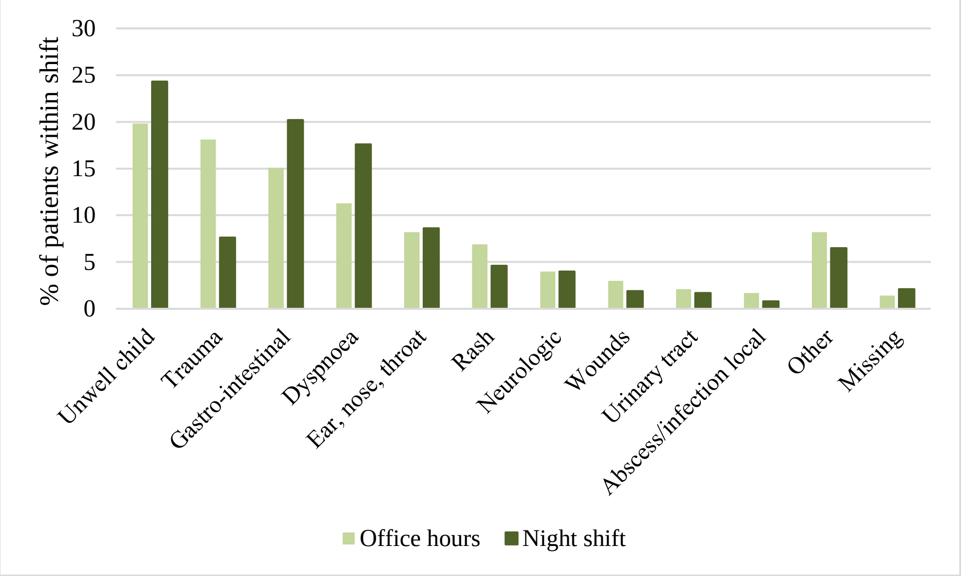

Confidential: For Review Only

Supplementary files

Supplementary table.1 Immediate life-saving interventions[a]
1. Airway and breathing support, including intubation or emergent non-invasive positive pressure ventilation.
2. Electrical therapy, including defibrillation, emergent cardioversion, or external pacing.
3. Procedures, including chest needle decompression, pericardiocentesis, or open thoracotomy.
4. Hemodynamic support, including significant intravenous fluid resuscitation in the setting of hypotension, blood administration, or control of major bleeding.
5. Emergency medications, including naloxone, dextrose, atropine, adenosine, epinephrine, or vasopressors

[a]Adapted from Lee et. al.(24)

Supplementary table.2 Missing vital signs and imputation model[a]

Vital sign	Missing
Heartrate	42.7%
Respiratory rate	52.4%
Temperature	22.5%
Oxygen saturation	44.9%

[a] Missing vital signs were imputed using a multiple imputation model including age, vital signs, MTS urgency, presenting problem and disposition after the ED. This imputation process resulted in twenty-five datasets on which statistical analyses were performed and pooled for a final result.(25) Imputation was performed by using the MICE imputation package in R, version 2.15.2.

Confidential: For Review Only

Supplementary table 3. Presenting problems				
N (%)	**Office hours (n=50.417)**	**Evening shift (n=36.429)**	**Night shift (n=14.138)**	**Day shift weekend (n=18.220)**
**Unwell child**	10.001 (19.8)	6.968 (19.1)	3.439 (24.3)	3.755 (20.6)
**Trauma**	9.144 (18.1)	6.951 (19.1)	1.075 (7.6)	2.950 (16.2)
**Gastro-intestinal**	7.613 (15.1)	5.274 (14.5)	2.860 (20.2)	2.470 (13.6)
**Dyspnoea**	5.686 (11.3)	3.822 (10.5)	2.494 (17.6)	1.947 (10.7)
**ENT**[a]	4.146 (8.2)	3.183 (8.7)	1.214 (8.6)	1.663 (9.1)
**Rash**	3.486 (6.9)	2.649 (7.3)	657 (4.6)	1.757 (9.6)
**Neurologic**	2.028 (4.0)	1.235 (3.4)	560 (4.0)	552 (3.0)
**Wounds**	1.536 (3.0)	1.685 (4.6)	274 (1.9)	760 (4.2)
**Urinary tract**	1.083 (2.1)	776 (2.1)	234 (1.7)	409 (2.2)
**Abscess/infection local**	842 (1.7)	517 (1.4)	113 (0.8)	347 (1.9)
**Other**	4.152 (8.2)	2.896 (7.9)	920 (6.5)	1.299 (7.1)
**Missing**	690 (1.4)	462 (1.3)	298 (2.1)	311 (1.7)

[a]ENT = Ear Nose Throat

Confidential: For Review Only

Supplementary table 4. Management adjusted regression analyses, per hospital					
	**Erasmus MC - Sophia Children's Hospital, Rotterdam the Netherlands**	**Maasstad Hospital, Rotterdam, the Netherlands**	**St. Mary's hospital Imperial College Healthcare NHS Trust, London, United Kingdom**	**Hospital Prof. Dr. Fernando Fonseca, Lisbon, Portugal**	**General Hospital, Vienna, Austria**
**Total n=119.204**	n = 18.590	n = 10.583	n = 15.556	n = 53.175	n = 21.300
**Lab**					
- Evening shift	0.76 (0.70 – 0.83)*	0.84 (0.73 – 0.95)*	0.73 (0.64 – 0.83)*	1.02 (0.96 – 1.08)	0.66 (0.61 – 0.71)*
- Night shift	0.82 (0.72 – 0.92)*	0.89 (0.75 - 1.06)	0.68 (0.58 – 0.81)*	1.07 (0.99 – 1.16)	0.81 (0.73 – 0.90)*
- Day shift weekend	0.89 (0.81 – 0.99)*	0.85 (0.72 – 1.01)	0.68 (0.58 – 0.81)*	1.13 (1.04 – 1.22)*	0.73 (0.67 – 0.80)*
**Imaging**					
- Evening shift	0.70 (0.64 – 0.76)*	1.90 (1.70 – 2.13)*	0.74 (0.66 – 0.83)*	0.87 (0.83 – 0.92)*	0.55 (0.48 – 0.63)*
- Night shift	0.53 (0.46 – 0.62)*	0.96 (0.79 – 1.18)	0.49 (0.41 – 0.59)*	0.71 (0.66 – 0.77)*	0.55 (0.45 – 0.68)*
- Day shift weekend	0.78 (0.70 – 0.87)*	1.82 (1.58 – 2.01)*	0.76 (0.66 – 0.89)*	0.86 (0.80 – 0.92)*	0.93 (0.80 – 1.08)
**Treatment**					
- Evening shift	1.11 (1.02 – 1.20)*	0.75 (0.66 – 0.86)*	1.36 (1.25 – 1.47)*	0.96 0.91 – 1.02	1.37 (1.26 – 1.50)*
- Night shift	1.32 (1.17 – 1.49)*	1.06 (0.89 – 1.27)	1.60 (1.45 – 1.78)*	1.68 (1.57 – 1.80)*	2.41 (2.14 – 2.71)*
- Day shift weekend	1.08 (0.98 – 1.19)	0.89 (0.75 – 1.05)	1.33 (1.20 – 1.47)*	1.08 (1.01 – 1.16)*	1.26 (1.13 – 1.41)*
**Hospital admission**					
- Evening shift	1.22 (1.12 – 1.34)*	1.06 (0.94 – 1.20)	0.92 (0.80 – 1.05)	1.05 (0.95 – 1.16)	0.89 (0.76 – 1.04)
- Night shift	1.48 (1.30 – 1.68)*	1.45 (1.23 – 1.72)*	0.96 (0.81 – 1.13)	1.64 (1.47 – 1.83)*	1.05 (0.85 – 1.30)
- Day shift weekend	1.13 (1.01 – 1.26)*	1.04 (0.89 – 1.21)	0.71 (0.59 – 0.85)*	1.06 (0.93 – 1.21)	1.27 (1.06 – 1.52)*

*P-value ≤0.05. If not stated: p-value >0.05

Confidential: For Review Only

BMJ Paediatrics Open

Management of children visiting the emergency department during out-of-office hours: an observational study

Journal:	*BMJ Paediatrics Open*
Manuscript ID	bmjpo-2020-000687.R1
Article Type:	Original research
Date Submitted by the Author:	07-May-2020

Complete List of Authors:	Schinkelshoek, Gina; Erasmus MC Sophia Children Hospital<br>Borensztajn, Dorine; Erasmus MC Sophia Children Hospital, Department of Pediatrics<br>Zachariasse, Joany; Erasmus MC Sophia Children Hospital, Department of Pediatrics<br>Maconochie, Ian; Imperial College Healthcare NHS Trust, Paediatric Emergency Department<br>Alves, Claudio; Professor Doctor Fernando Fonseca Hospital, General Paediatrics<br>Freitas , Paulo ; Professor Doctor Fernando Fonseca Hospital, Intensive Care Unit<br>Smit, Frank; Maasstad Hospital, Paediatrics<br>van der Lei, Johan; Erasmus Medical Center, Department of Medical Informatics<br>Steyerberg, Ewout; Erasmus Medical Center, Public Health<br>Greber-Platzer, Susanne; Medical University of Vienna, Division of Pediatric Pulmology, Allergology and Endocrinology, Department of Pediatrics and Adolescent Medicine<br>moll, Henriette; Erasmus MC Sophia Children Hospital, Department of Pediatrics

Keywords:	Accident & Emergency, General Paediatrics, Paediatric Practice

SCHOLARONE™
Manuscripts

I, the Submitting Author has the right to grant and does grant on behalf of all authors of the Work (as defined in the below author licence), an exclusive licence and/or a non-exclusive licence for contributions from authors who are: i) UK Crown employees; ii) where BMJ has agreed a CC-BY licence shall apply, and/or iii) in accordance with the terms applicable for US Federal Government officers or employees acting as part of their official duties; on a worldwide, perpetual, irrevocable, royalty-free basis to BMJ Publishing Group Ltd ("BMJ") its licensees and where the relevant Journal is co-owned by BMJ to the co-owners of the Journal, to publish the Work in this journal and any other BMJ products and to exploit all rights, as set out in our licence.

The Submitting Author accepts and understands that any supply made under these terms is made by BMJ to the Submitting Author unless you are acting as an employee on behalf of your employer or a postgraduate student of an affiliated institution which is paying any applicable article publishing charge ("APC") for Open Access articles. Where the Submitting Author wishes to make the Work available on an Open Access basis (and intends to pay the relevant APC), the terms of reuse of such Open Access shall be governed by a Creative Commons licence – details of these licences and which Creative Commons licence will apply to this Work are set out in our licence referred to above.

Other than as permitted in any relevant BMJ Author's Self Archiving Policies, I confirm this Work has not been accepted for publication elsewhere, is not being considered for publication elsewhere and does not duplicate material already published. I confirm all authors consent to publication of this Work and authorise the granting of this licence.

Confidential: For Review Only

MANAGEMENT OF CHILDREN VISITING THE EMERGENCY DEPARTMENT DURING OUT-OF-OFFICE HOURS: AN OBSERVATIONAL STUDY

Gina Schinkelshoek[1*], Dorine M. Borensztajn[1*], Joany M. Zachariasse[1], Ian K. Maconochie[2], Claudio F. Alves[3], Paulo Freitas[4], Frank J. Smit[5], Johan van der Lei[6], Ewout W. Steyerberg[7], Susanne Greber-Platzer[8], Henriëtte A. Moll[1]

Affliations:
[1]Erasmus MC – Sophia Children's Hospital, Department of General Paediatrics, Rotterdam, The Netherlands.
[2]Imperial College NHS Healthcare Trust, Department of Paediatric Accident and Emergency, London, United Kingdom.
[3]Hospital Prof. Dr. Fernando Fonseca, Department of Paediatrics, Lisbon, Portugal.
[4]Hospital Prof. Dr. Fernando Fonseca, Intensive Care Unit, Lisbon, Portugal.
[5]Maasstad Hospital, Department of Paediatrics, Rotterdam, the Netherlands.
[6]Erasmus MC- University Medical Centre Rotterdam, Department of Medical Informatics, Rotterdam, The Netherlands.
[7]Leiden University Medical Centre, Department of Biomedical Data Sciences, Leiden, The Netherlands.
[8]Medical University Vienna, Department of Paediatrics and Adolescent Medicine, Vienna, Austria.
*Gina Schinkelshoek and Dorine M. Borensztajn contributed equally to this paper.

Corresponding Author:
Prof. Dr. H.A. Moll
Department of General Paediatrics
Erasmus MC – Sophia Children's Hospital
P.O. Box 2060, 3000 CB, Rotterdam, The Netherlands.
Telephone: +31 10 703 61 96
Fax: +31 10 703 66 85
Email: H.A.Moll@erasmusmc.nl

Word count:
2641 words

Confidential: For Review Only

What is already known on this topic:

Concerns exist that emergency care may differ during out-of-office hours and office hours. There has been growing attention for paediatric emergency care at the emergency department recently. However, little is known about children visiting the emergency department during out-of-office hours.

What this study adds:

Children visiting the emergency department during the night were more seriously ill than during office hours. Fewer diagnostic tests, more treatment and more hospital admissions were reported during the night, which could not be explained by correcting for illness severity.

Confidential: For Review Only

ABSTRACT

Background
The aim was to study the characteristics and management of children visiting the Emergency Department (ED) during out-of-office hours.
Methods
We analysed electronic health record data from 119.204 children visiting one of five EDs in four European countries. Patient characteristics and management (diagnostic tests, treatment, hospital admission and Paediatric Intensive Care Unit admission) were compared between children visiting during office hours and evening shifts, night shifts and weekend day shifts. Analyses were corrected for age, gender, MTS urgency, abnormal vital signs, presenting problem and hospital.
Results
Patients presenting at night were younger (median (IQR) age: 3.7 (1.4 – 8.2) years versus 4.8 (1.8 – 9.9)), more often classified as high urgent (16.3% versus 9.9%) and more often had ≥ 2 abnormal vital signs (22.8% versus 18.1%), compared to office hours. After correcting for disease severity, laboratory and radiological tests were less likely to be requested (aOR: 0.82, 95% CI: 0.78 – 0.86 and aOR: 0.64, 95% CI: 0.60 – 0.67 respectively); treatment was more likely to be undertaken (aOR: 1.56, 95% CI: 1.49 – 1.63) and patients were more likely to be admitted to the hospital (aOR: 1.32, 95% CI: 1.24 -1.41) at night. Patterns in management during out-of-office hours were comparable between the different hospitals.
Conclusions
Children visiting during the night are relatively more seriously ill, highlighting the need for optimal emergency care 24 hours a day. Further research is needed to explain the differences in management during the night and how these differences affect patient outcome.

Confidential: For Review Only

INTRODUCTION

Concerns exist that emergency care may differ during out-of-office hours, i.e. evening, night and/or weekend shifts, compared to office hours. For example, higher mortality rates were found during weekend visits in adult patients with sepsis presenting to the Emergency Department (ED).(1) Some suggest that worse patient outcome during out-of-office hours can, partly, be explained by higher illness severity and not by time of arrival as independent factor.(2, 3) When looking at the paediatric population, higher mortality rates were observed among children admitted to the PICU during out-of-office hours.(4) However, other studies analysing PICU patients are in conflict with this finding (5-9).

Over the past few years, increasing attention has been paid to paediatric emergency care, leading to different guidelines and standards for children visiting the ED.(10-14) These guidelines aim to achieve optimal utilization of available resources, optimize patient outcome and thereby improve quality of care. A recent study reported that main standards of care are fulfilled in European EDs, but some areas of improvement still exist.

However, little research has focused specifically on children visiting the ED during out-of-office hours. Recently, variation in paediatric ED utilization patterns and the presentation of these children by shift of arrival has been reported.(15) But this study was a single centre study, which may limit the generalizability. Moreover, no study has analysed resource utilization at paediatric EDs during out-of-office hours, except for studies analysing children with a specific presenting problem.(16-18)

It is important to obtain insight into the characteristics and management of the whole paediatric patient population attending the ED with its variable presenting problems, as this may be helpful in optimizing guidelines and resource allocation, and thereby in improving the quality of care on a 24 hours a day basis.

Confidential: For Review Only

The aim of this study was to compare patient characteristics and management of paediatric patients visiting the ED during out-of-office hours to office hours, in four different European countries.

Confidential: For Review Only

METHODS

Study design, setting, patients

This study is part of the TrIAGE project (Triage Improvement Across General Emergency departments for paediatric patients), a prospective observational study.(19) This project captured routinely collected, standardized, electronic health record data of all non-scheduled ED visits of children <16 years in five different hospitals in four different countries. The five participating hospitals, selected by convenience sampling, were: Erasmus Medical Centre, the Netherlands; Maasstad Hospital, the Netherlands; St. Mary's hospital Imperial College Healthcare NHS Trust, United Kingdom; Hospital Prof. Dr. Fernando Fonseca, Portugal; Vienna General Hospital, Austria. The enrolment period varied from 8 to 36 months between 2012 and 2015.

At the beginning of the study, a minimum dataset of variables for collection was defined. If necessary, entries in the electronic health data were added or modified. Nurses at the ED were informed about the study and encouraged to fill in the entire medical records, including vital signs, which is in principle routine data. During the study this routinely collected data was documented in the electronic, medical records by nurses at the ED. Data completeness and accuracy of the data was reviewed using a checklist of quality control. Sometimes, data was completed by information or vital signs from medical records documented by physicians at the ED. Coded data were extracted from the electronic hospital information systems and thereafter transferred to the principal investigator as an encrypted file. Careful data harmonization and quality checks were conducted. The requirement for informed consent was obtained by all hospitals and the study was approved by the medical ethical committees of all participating institutions.

Patient and Public Involvement statement

Confidential: For Review Only

Patients or the public were not involved in this research.

Definitions

Office hours versus out-of-office hours

Office hours were defined as Monday until Friday, between 08:00 am and 05:59 pm, also referred to as day shift week. We compared office hours, as reference shift, to out-of-office hours, divided into evening shift, night shifts and day shifts during the weekend. Evening shifts were defined as Monday until Sunday between 6:00 pm and 10:59 pm and night shifts as Monday until Sunday between 11:00 pm and 07:59 am, based on previous literature. (5, 9, 16) Weekend day shifts were defined as Saturday and Sunday between 08:00 am and 5:59 pm.

Patient characteristics

For this study, the following data were extracted from the database: age, gender, time and date of arrival, Manchester Triage System (MTS) urgency, vital signs and presenting symptom (based on the MTS flowchart). The MTS urgency consists of five categories: emergent, very urgent, urgent, standard and non-urgent.(20-22) These categories were divided in three groups: high urgent (emergent or very urgent, <10 minutes waiting time), urgent (urgent, <60 minutes waiting time), low urgent (standard or non-urgent, ≥ 60 minutes waiting time) . Vital signs included heart rate, respiratory rate, oxygen saturation and temperature. Vital signs were considered abnormal based on the Advanced Paediatric Life Support (APLS) reference values, with fever defined as temperature ≥38.5ºC.(23) The MTS flowchart was used to create subgroups of presenting problems. These subgroups, based on a previous study (24), included medical (respiratory, ear, nose and throat (ENT) problems, gastro-intestinal problems, neurological or psychiatric problems, general malaise, uro- or gynaecological problems, dermatologic, others) and trauma (trauma or muscular and wounds) (supplementary table 2) .

https://mc.manuscriptcentral.com/bmjpo

Confidential: For Review Only

Management

Management was defined as diagnostics, (laboratory tests and imaging), treatment at the ED and hospital admission directly after ED visit. Laboratory tests were divided into: blood tests, including cultures; and urine tests, including cultures. Imaging was divided into: x-ray, ultrasound and Computed Tomography (CT).

Treatment was divided into: oral medication, inhalation medication, intravenous (IV) medication or IV fluids and Immediate Lifesaving Interventions (ILI) (see supplementary table 1 (25)).

In the multivariable regression models, we defined laboratory diagnostics as any form of laboratory tests, radiological diagnostics as any form of imaging and treatment as any form of treatment.

Hospital admission included patients who were admitted to the general ward or to the PICU. PICU admission was defined as admission to the PICU directly from the ED.

Statistical analysis

In descriptive statistics of patient characteristics continuous, skewed variables are shown as median with interquartile range (IQR). Nominal and ordinal variables are shown as number of patients with percentage within shift. Nominal and ordinal variables considering management are shown as percentages within shift, with inter-hospital ranges.

In multivariable regression analyses differences in management were compared between office hours and evening, night and weekend day shifts. To study the independent role of shift of arrival, the regression analyses were adjusted for age, gender, MTS urgency, abnormal vital signs, presenting problem and hospital. We performed stratified analyses, by conducting the

Confidential: For Review Only

adjusted regression analyses for each hospital separately, to provide insight into inter-hospital differences.

Missing vital signs were imputed using a multiple imputation model. (table 1 and supplementary figure 1 (26))

Results were considered statistically significant at a P-value of ≤ 0.05.

Initial analyses were performed with SPSS software, version 21 (IBM SPSS statistics, IBM corporation, Armonk, NY).

Confidential: For Review Only

RESULTS

Patient population

In total, 119,209 patients, ≤16 years, visited the participating EDs. Five patients were excluded owing to missing arrival time or date, leaving 119,204 patients for analysis, with 50,417 (42.3%) of these patients who visited the ED during office hours versus 68,787 (57.7%) during out-of-office hours. Of those, 18,220 (15.3%) visited during weekend day shifts, 36,429 (30.6%) during the evening and 14,138 (11.9%) during the night.

Patients who visited the ED during the night were younger, were more often classified by MTS as high urgent, and more often had two or more abnormal vital signs (table 2).

During office hours, general malaise (20.0%), trauma or muscular (19.4%) and gastro-intestinal problems (15.1%) were the most common presenting problems (figure 1). During the night, relatively more general malaise (24.6%), gastro-intestinal problems (20.2%), respiratory (17.6%) and fewer trauma cases (8.2%) were observed. Presenting problems during the evening and weekend day shifts were similar compared to office hours. (supplementary table 3)

Management

During out-of-office hours, fewer diagnostic tests, more treatment and more hospital and PICU admissions were conducted. (table 3)

During the evening and weekend day shift, fewer urine and blood tests were performed.

During the evening, night and weekend day shifts, imaging was performed less often, except for X-ray during the evening shift. Treatment with oral, inhaled and intravenous medication were given more often, especially during the night. Also, during the night, slightly more immediate lifesaving interventions were seen, whilst more hospital and ICU admissions were observed.

Confidential: For Review Only

In multivariable regression analyses, the results were adjusted for age, gender, urgency, abnormal vital signs and presenting problem (medical or trauma) and hospital. (table 4) Laboratory and imaging diagnostics were significantly less likely to be performed during the evening and night shifts. Also, patients were significantly more likely to receive treatment during all out-of-office hours shifts and more likely to be admitted to the hospital during the night shift. After adjusting, no association was found between PICU admission and out-of-office hours visits.

Inter-hospital analyses

Hospital characteristics are shown in table 5. Some inter-hospital variation in management was observed. (table 2) The multivariable regression analyses, adjusted for age, gender, urgency level, abnormal vital signs and presenting problem, were performed for each hospital separately in stratified analyses. Overall patterns in management during out-of-office hours were comparable between the different hospitals, with some variation remaining. (supplementary table 4)

Confidential: For Review Only

DISCUSSION

In this large observational study of 119,204 visits in five EDs in Europe, we demonstrate differences acuity level and management of children visiting the ED during out-of-office hours compared to office hours.

We found that children visiting during the night were relatively more seriously ill. Secondly, after correcting for patient characteristics, illness severity and hospital, we observed fewer laboratory and radiological tests whilst children were more likely to receive treatment at the ED and to be admitted to the hospital during especially the night. No association was found between out-of-office hours visits and PICU admission, after statistically correcting for disease severity. Overall, the trends in out-of-office hours care were comparable between the different hospitals.

In a recent study, ED utilization patterns of paediatric patients through the day was analysed.(15) The distribution of visits over the evening and night shifts was quite similar to our results. Also, in line with our results, they observed that children during the night are relatively more ill. So, although the proportion of patients visiting during the night is the smallest, the acuity of this group is the highest, which shows the importance of the same quality of care on a 24 hours a day basis. In contrast to our results, no association between shift of arrival and hospital admission was reported. However, this was a single centre study, which limits the generalizability.

Variation in management during out-of-office hours, most pronounced during the night, could not be explained by illness severity. Reducing variation in care could result in lower resource utilization and costs, and could improve the use of time, space and staff. (27) To obtain more standardized care, also during out-of-office hours, it is essential to understand which factors contribute to the differences we observed.

https://mc.manuscriptcentral.com/bmjpo

Confidential: For Review Only

At first, different levels of staff during the night might influence management. Several studies found differences in resource use (28-32) and admission rates (33) depending on physician background and experience.

Secondly, availability of resources during night shifts, such as imaging resources or the availability of specialized radiologists to assess these results during the night, might contribute to the lower use of imaging during the night. (34)

Our results raise the question whether these higher treatment and admission rates, and the lower use of diagnostic imaging during the night, might have been unwarranted and contribute to unnecessary costs. Earlier research reported that higher resource utilization is related to higher costs, but not to lower admission rates. (35-37) One of the hospitals in our study showed no difference in diagnostics during the night, in contrast to the other hospitals, but still showed higher admission rates compared to office hours.

We speculate that our results might also partly be explained by a more pragmatic approach of the patient during the night, resulting in fewer diagnostic tests, more treatment and more hospital admissions. Previous literature did report higher rates of antibiotic prescriptions (16) and recommendations to admit to the hospital (38), in children visiting the ED during the night. Additionally, parental demand might contribute to these results. (39) Furthermore, in contrast to general hospital admission, the higher rates of PICU admission during the night, could be explained by illness severity and case mix. The indications for PICU admissions are more standardized compared to indications for general hospital and therefore less influenced by non-patient factors.

These results point to the need for further research to explain the differences in clinical decisions during the night, by assessing differences in standards of care during out-of-office hours, such as availability of experienced staff and availability of diagnostics and equipment.(14) Moreover, it is important to obtain insight in how these differences in

Confidential: For Review Only

management affect patient outcome, for example rehospitalisation, morbidity and 30-day mortality. Implementing guidelines for management of children with specific conditions, could help to reduce variation in management within and between hospitals, which optimize resource allocation and costs. Moreover, these guidelines could allow for better comparisons between the hospitals in further research.

Other studies on the topic of out-of-office hours care, including adult patients at the ED and paediatric patients at the PICU, described worse patient outcome during the night and evening, represented by higher mortality rates. (1, 4, 40) We did not use mortality as the main outcome as the mortality in our dataset was very low (16 patients, 0.01%), which is in line with previous literature. (41, 42)

Strengths and limitations

A major strength of this study is the large number of patients from different centres, including both university as teaching hospitals. This increases the generalizability of the results. Moreover, we adjusted for multiple variables concerning illness severity to analyse the independent role of time of visit to management.

To appreciate the results, some limitations have to be considered. The enrolment period varied between the different hospitals. However, each hospital was followed for at least 8 months and in all hospitals a winter season was included to increase the comparability of the periods. Also, to our knowledge, there were no major changes or differences in for example vaccination status, occurrence of epidemics or other incidents during the total enrolment period (2012-2015).

Secondly, the analyses were based on routinely collected data. This results in some missing data, which we dealt with using multiple imputation for vital signs. Moreover, it is possible that there are more variables representing illness severity and influencing management, that were not included. These factors can be divided into patient- and hospital-related factors. Patient-

https://mc.manuscriptcentral.com/bmjpo

Confidential: For Review Only

related factors are e.g. comorbidity, socio-economic status or ethnic background. When looking at hospital-related factors, (experience level of) staff, resource availability and bed availability could possibly contribute to the differences in management. To deal with hospital-related differences, we adjusted our regression analyses for hospital. Furthermore, we performed the regression analyses for each hospital separately. These stratified analyses demonstrated that overall results were comparable between the hospitals, but some variation still remained. However, we did not have the specific information about the aforementioned hospital-level factors for each individual visit, to include in the analyses. It would be interesting to analyse the potential effects of these factors in future research.

Conclusion

Children visiting during the night are relatively more seriously ill than those visiting during office hours. This highlights the need for optimal emergency care on a 24 hours a day basis. Furthermore, differences in management are present depending on the time of ED visit. Especially during the night, diagnostic tests are performed less frequently, whilst treatment is initiated more often and more children are admitted to the hospital than during office hours. These patterns are not explained by illness severity. Further research should focus on exploring the reasons for these differences and how these differences affect patient outcome.

Confidential: For Review Only

FUNDING STATEMENT

This research received no specific grant from any funding agency in the public, commercial or not-for-profit sectors.

COMPETING INTERESTS

All authors have no conflicts of interest relevant to this article to disclose.

CONTRIBUTORSHIP STATEMENT

Gina Schinkelshoek: Conceptualization, data curation, formal analysis, investigation, writing original draft, review and editing.

Dorine M. Borensztajn: Conceptualization, data curation, formal analysis, investigation, writing original draft, review and editing.

Joany M. Zachariasse: Data curation, formal analysis, investigation, review and editing.

Susanne Greber-Platzer: Data curation, investigation, review and editing.

Claudio F. Alves: Data curation, investigation, review and editing.

Paulo Freitas: Data curation, investigation, review and editing.

Frank J. Smit: Data curation, investigation, review and editing.

Ian K. Maconochie: Data curation, investigation, review and editing.

Johan van der Lei: Conceptualization, data curation, review and editing.

Ewout W. Steyerberg: Data curation, review and editing.

Henriëtte A. Moll: Conceptualization, data curation, investigation, project administration, supervision, review and editing.

All authors approved the final manuscript as submitted and agree to be accountable for all aspects of work.

Confidential: For Review Only

DATA SHARING STATEMENT

Data from this study are available upon request to the corresponding author of the study (h.a.moll@erasmusmc.nl), subject to local rules and regulations.

Confidential: For Review Only

References

1. Powell ES, Khare RK, Courtney DM, Feinglass J. The weekend effect for patients with sepsis presenting to the emergency department. J Emerg Med. 2013 Nov;45(5):641-8.
2. Mikulich O, Callaly E, Bennett K, O'Riordan D, Silke B. The increased mortality associated with a weekend emergency admission is due to increased illness severity and altered case-mix. Acute Med. 2011;10(4):182-7.
3. Walker AS, Mason A, Quan TP, Fawcett NJ, Watkinson P, Llewelyn M, et al. Mortality risks associated with emergency admissions during weekends and public holidays: an analysis of electronic health records. Lancet. 2017 May 09.
4. Arias Y, Taylor DS, Marcin JP. Association between evening admissions and higher mortality rates in the pediatric intensive care unit. Pediatrics. 2004 Jun;113(6):e530-4.
5. Peeters B, Jansen NJ, Bollen CW, van Vught AJ, van der Heide D, Albers MJ. Off-hours admission and mortality in two pediatric intensive care units without 24-h in-house senior staff attendance. Intensive Care Med. 2010 Nov;36(11):1923-7.
6. McShane P, Draper ES, McKinney PA, McFadzean J, Parslow RC, Paediatric Intensive Care Audit N. Effects of out-of-hours and winter admissions and number of patients per unit on mortality in pediatric intensive care. J Pediatr. 2013 Oct;163(4):1039-44 e5.
7. Thabet FC, alHaffaf FA, Bougmiza IM, Bafaqih HA, Chehab MS, alMohaimeed SA. Off-Hours Admissions and Mortality in PICU Without 24-Hour Onsite Intensivist Coverage. J Intensive Care Med. 2018 Jan 1:885066618778824.
8. Hixson ED, Davis S, Morris S, Harrison AM. Do weekends or evenings matter in a pediatric intensive care unit? Pediatr Crit Care Med. 2005 Sep;6(5):523-30.
9. Numa A, Williams G, Awad J, Duffy B. After-hours admissions are not associated with increased risk-adjusted mortality in pediatric intensive care. Intensive Care Med. 2008 Jan;34(1):148-51.
10. American Academy of Pediatrics Committee on Pediatric Emergency M, American College of Emergency Physicians Pediatric C, Emergency Nurses Association Pediatric C. Joint policy statement--guidelines for care of children in the emergency department. Ann Emerg Med. 2009 Oct;54(4):543-52.
11. Gausche-Hill M, Wiebe RA. Guidelines for preparedness of emergency departments that care for children: A call to action. Annals of Emergency Medicine. [doi: 10.1067/mem.2001.114069]. 2001;37(4):389-91.
12. Remick K, Gausche-Hill M, Joseph MM, Brown K, Snow SK, Wright JL, et al. Pediatric Readiness in the Emergency Department. Pediatrics. 2018 Nov;142(5).
13. Facing the Future: Standards for children in emergency care settings. Royal College of Paediatrics and Child Health; 2018 [cited 2019 October]; Available from: https://www.rcpch.ac.uk/sites/default/files/2018-06/FTFEC%20Digital%20updated%20final.pdf.
14. Standards of Care for Children in Emergency Departments (Third version). International Federation for Emergency Medicine; 2012 [updated April 2019; cited 2019 October]; Available from: https://www.ifem.cc/wp-content/uploads/2019/06/Standards-of-Care-for-Children-in-Emergency-Departments-V3-2019.pdf.
15. Almubarak H, Meckler G, Doan Q. Factors and outcomes associated with paediatric emergency department arrival patterns through the day. Paediatr Child Health. 2019 Aug;24(5):323-9.
16. Roggen I, van Berlaer G, Gordts F, Pierard D, Hubloue I. Acute sore throat in children at the emergency department: best medical practice? Eur J Emerg Med. 2015 Oct;22(5):343-7.

Confidential: For Review Only

17. Johnson EK, Graham DA, Chow JS, Nelson CP. Nationwide emergency department imaging practices for pediatric urolithiasis: room for improvement. J Urol. 2014 Jul;192(1):200-6.
18. Daverio M, Bressan S, Gregori D, Babl FE, Mackay MT. Patient and Process Factors Associated With Type of First Neuroimaging and Delayed Diagnosis in Childhood Arterial Ischemic Stroke. Acad Emerg Med. 2016 Sep;23(9):1040-7.
19. Zachariasse JM, Nieboer D, Maconochie IK, Smit FJ, Alves CF, Greber-Platzer S, et al. Development and validation of a Paediatric Early Warning Score for use in the emergency department: a multicentre study. Accepted for publication in Lancet Child and Adolescent Health. 2020.
20. De Caluwé R, Manchester Triage G. Triage voor de spoedeisende hulp: Elsevier Gezondheidszorg; 2007.
21. van Veen M, Steyerberg EW, van't Klooster M, Ruige M, van Meurs AHJ, van der Lei J, et al. The Manchester triage system: improvements for paediatric emergency care. Emergency Medicine Journal. 2012;29(8):654-9.
22. Mackway-Jones K, Marsden J, Windle J. Emergency Triage: Manchester Triage Group. Third ed. Oxford, United Kingdom: John Wiley & Sons; 2013.
23. Turner NM. Advanced paediatric life support : de Nederlandse editie. Vijfde druk. ed. Houten :: Bohn Stafleu van Loghum; 2017.
24. Zachariasse JM, Seiger N, Rood PP, Alves CF, Freitas P, Smit FJ, et al. Validity of the Manchester Triage System in emergency care: A prospective observational study. PLoS One. 2017;12(2):e0170811.
25. Lee JY, Oh SH, Peck EH, Lee JM, Park KN, Kim SH, et al. The validity of the Canadian Triage and Acuity Scale in predicting resource utilization and the need for immediate life-saving interventions in elderly emergency department patients. Scand J Trauma Resusc Emerg Med. 2011 Nov 3;19:68.
26. Donders AR, van der Heijden GJ, Stijnen T, Moons KG. Review: a gentle introduction to imputation of missing values. J Clin Epidemiol. 2006 Oct;59(10):1087-91.
27. Neuman MI, Chiang VW. Variation in pediatric care at US hospitals. Pediatrics. 2013 Aug;132(2):369-70.
28. Pitts SR, Morgan SR, Schrager JD, Berger TJ. Emergency department resource use by supervised residents vs attending physicians alone. Jama. 2014 Dec 10;312(22):2394-400.
29. White AL, Armstrong PA, Thakore S. Impact of senior clinical review on patient disposition from the emergency department. Emerg Med J. 2010 Apr;27(4):262-5, 96.
30. Hampers LC, Faries SG. Practice variation in the emergency management of croup. Pediatrics. 2002 Mar;109(3):505-8.
31. Melo MR, Ferreira-Magalhaes M, Flor-Lima F, Rodrigues M, Severo M, Almeida-Santos L, et al. Dedicated Pediatricians in Emergency Department: Shorter Waiting Times and Lower Costs. PLoS One. 2016;11(8):e0161149.
32. Knapp JF, Simon SD, Sharma V. Variation and trends in ED use of radiographs for asthma, bronchiolitis, and croup in children. Pediatrics. 2013 Aug;132(2):245-52.
33. Scott-Jupp R, Carter E, Brown N. Effects of consultant residence out-of-hours on acute paediatric admissions. Arch Dis Child. 2020 Jan 14.
34. Fullerton K, Depinet H, Iyer S, Hall M, Herr S, Morton I, et al. Association of Hospital Resources and Imaging Choice for Appendicitis in Pediatric Emergency Departments. Acad Emerg Med. 2017 Apr;24(4):400-9.
35. Kharbanda AB, Hall M, Shah SS, Freedman SB, Mistry RD, Macias CG, et al. Variation in resource utilization across a national sample of pediatric emergency departments. J Pediatr. 2013 Jul;163(1):230-6.

36. Florin TA, French B, Zorc JJ, Alpern ER, Shah SS. Variation in emergency department diagnostic testing and disposition outcomes in pneumonia. Pediatrics. 2013 Aug;132(2):237-44.
37. Bourgeois FT, Monuteaux MC, Stack AM, Neuman MI. Variation in emergency department admission rates in US children's hospitals. Pediatrics. 2014 Sep;134(3):539-45.
38. El Khoury AC, Durden E, Ma L, Markson LE, Lee AW, Duan Y, et al. Perception and management of fever in infants up to six months of age: a survey of US pediatricans. BMC Pediatr. 2010 Dec 22;10:95.
39. Pachter LM, Ludwig S, Groves A. Night people: utilization of a pediatric emergency department during the late night. Pediatr Emerg Care. 1991 Feb;7(1):12-4.
40. Duvald I, Moellekaer A, Boysen MA, Vest-Hansen B. Linking the severity of illness and the weekend effect: a cohort study examining emergency department visits. Scand J Trauma Resusc Emerg Med. 2018 Sep 5;26(1):72.
41. Lopez E, Udaondo J, Olabarri M, Martinez-Indart L, Benito J, Mintegi S, et al. Mortality in Spanish pediatric emergency departments: a 5-year multicenter survey. Eur J Emerg Med. 2017 Dec;24(6):392-7.
42. O'Malley P, Barata I, Snow S, American Academy of Pediatrics Committee on Pediatric Emergency M, American College of Emergency Physicians Pediatric Emergency Medicine C, Emergency Nurses Association Pediatric C. Death of a child in the emergency department. Pediatrics. 2014 Jul;134(1):e313-30.

Confidential: For Review Only

Table 1. Missing vital signs and imputation model[a]

Vital sign	Missing<br>% of total patients (inter-hospital range)
**Heartrate**	42.7 (19.5 – 62.9)
**Respiratory rate**	52.4 (23.5 – 87.4)
**Oxygen saturation**	44.9 (19.7 – 66.4)
**Temperature**	22.5 (5.9 – 58.2)

[a]Missing vital signs were imputed using a multiple imputation model including age, vital signs, MTS urgency, presenting problem and disposition after the ED. This imputation process resulted in twenty-five datasets on which statistical analyses were performed and pooled for a final result.(26) Imputation was performed by using the MICE imputation package in R, version 2.15.2.

Table 2. Patient characteristics

(Total, n=119,204)	**Office hours[a]** **(n=50,417)**	**Evening shift** **(n=36,429)**	**Night shift** **(n=14,138)**	**Day shift weekend** **(n=18,220)**
**Median age, yrs *(IQR[b])***	4.8 (1.8 – 9.9)	4.5 (1.8 – 9.5)	3.7 (1.4 – 8.2)	4.2 (1.7 – 8.8)
**Gender, *n (%)***				
- **Male**	27,150 (53.9)	19,698 (54.1)	7,566 (53.5)	9,956 (54.6)
**MTS, *n (%)***				
- **High urgent**	4,963 (9.9)	4,136 (10.7)	2,302 (16.3)	1,683 (9.2)
- **Urgent**	13,300 (26.4)	10,796 (29.6)	3,655 (25.9)	4,866 (26.7)
- **Low urgent**	31,478 (61.7)	21,276 (58.4)	7,892 (55.8)	11,366 (62.4)
- **Missing**	676 (1.3)	452 (1.2)	289 (2.0)	305 (1.7)
**Vital signs, *n (%)***				
- **Normal**	18,734 (37.2)	12,903 (35.4)	4,812 (34.0)	6,553 (36.0)
- **1 Abnormal**	22,545 (44.7)	15,947 (43.8)	6,100 (43.1)	8,119 (44.6)
- **2 or more abnormal**	9,139 (18.1)	7,578 (20.8)	3,227 (22.8)	3,548 (19.5)

[a]Office hours = Day shift week
[b]IQR = Interquartile range
[c]MTS = Manchester Triage System

Confidential: For Review Only

Table 3. Management (diagnostics, treatment, hospital admission and PICU admission), descriptive

Total, n = 119,204	**Office hours[a] (n=50,417)**	**Evening shift (n=36,429)**	**Night shift (n=14,138)**	**Day shift weekend (n=18,220)**
**Mean % (inter-hospital range)**				
**<u>Laboratory diagnostics</u>**				
**Any lab test (n=119,204)**	22.2 (11.7 – 40.6)	18.6 (9.2 – 33.0)	21.5 (9.8 – 40.0	20.2 (8.0 – 33.3)
**Urine tests (n=50,473)[b]**	20.2 (11.0 - 23.5)	17.1 (8.1 – 22.3)	20.2 (13.9 – 23.2)	16.6 (7.7 – 19.6)
**Blood tests (n=66,029)[b]**	21.9 (11.7 – 26.7)	15.2 (9.2 – 20.3)	20.4 (9.8 - 30.5)	17.2 (7.9 – 23.5)
**<u>Imaging diagnostics</u>**				
**X-ray (n=119,204)**	19.5 (6.0 - 24.7)	19.4 (3.7 – 40.9)	12.1 (4.3 – 17.1)	17.4 (5.5 -41.2)
**Ultrasound (n=119,204)**	2.7 (1.5 - 6.0)	1.7 (0.5 - 2.9)	1.2 (0.5 - 4.1)	2.2 (0.6 - 4.2)
**CT (n=119,204)**	1.2 (0.7 - 2.5)	1.0 (0.6 - 2.4)	0.9 (0.4 - 2.3)	1.0 (0.5 - 2.1)
**<u>Treatment</u>**				
**Oral medication (n=108,621)[b]**	13.9 (8.0 – 34.3)	17.2 (8.6 – 41.8)	23.5 (14.9 – 45.1)	16.5 (8.2 – 39.7)
**Inhaled medication (n=119,204)**	6.5 (3.6 - 8.7)	6.5 (3.5 – 9.0)	12.0 (6.0 – 15.4)	6.9 (3.5 – 9.8)
**IV[c] medication or fluids (n=119,204)**	7.3 (4.1 - 14.0)	7.0 (4.2 - 11.3)	11.3 (4.6 - 19.4)	7.8 (3.4 - 12.0)
**ILI[d] (n=119,204)**	0.3 (0.0 – 1.1)	0.3 (0.0 -1.2)	0.5 (0.0 -1.9)	0.4 (0.1 -1.2)
**<u>Hospital admission</u> (n=119,204)**				
**Yes**	9.7 (4.3 - 22.6)	10.2 (5.0 – 24.2)	14.3 (8.8 – 38.4)	9.9 (4.6 – 22.4)
**<u>PICU admission</u> (n=119,204)**				
**Yes**	0.6 (0.1 – 2.3)	0.5 (0.1 -2.8)	0.8 (0.2 - 4.7)	0.6 (0.0 - 2.7)

[a]Office hours = Day shift week
[b]Not all variables were complete for all hospitals, making the total number less than 119.204.
[c] IV = Intravenous
[d] ILI = Immediate Lifesaving Interventions

Confidential: For Review Only

Table 4. The association between management (lab, imaging, treatment, hospital and PICU[a] admission) and time of arrival at the ED, adjusted regression analyses

Total, n = 119,204<br>aOR[b] (95% CI)	**Office hours[c] (n=50417)**	**Evening shift (n=36,429)**	**Night shift (n=14,138)**	**Day shift weekend (n=18,220)**
**Lab**	-<br>-	0.79<br>(0.76 – 0.82) *	0.82<br>(0.78 – 0.86) *	0.92<br>(0.87 – 0.96) *
**Imaging**	-<br>-	0.93<br>(0.89 – 0.96) *	0.64<br>(0.60 – 0.67) *	0.99<br>(0.94 – 1.04)
**Treatment**	-<br>-	1.11<br>(1.07 – 1.15) *	1.56<br>(1.49 – 1.63) *	1.17<br>(1.12 – 1.22) *
**Hospital admission**	-<br>-	1.06<br>(1.00 – 1.11) *	1.32<br>(1.24 – 1.41) *	1.05<br>(0.99 – 1.12)
**PICU[a] admission**	-<br>-	1.15<br>(0.95 – 1.42)	0.92<br>(0.71 – 1.19)	1.19<br>(0.93 – 1.52)

[a] PICU = Paediatric Intensive Care Unit
[b]aOR = Adjusted Odds Ratio. Adjusted for age, gender, urgency, vital signs, presenting problem and hospital.
[c] Office hours (= day shift week) as reference shift.
* P-value ≤0.05. If not stated: p-value >0.05

Confidential: For Review Only

Table 5. Hospital characteristics

	Erasmus MC - Sophia Children's Hospital, Rotterdam the Netherlands	Maasstad Hospital, Rotterdam, the Netherlands	St. Mary's hospital Imperial College Healthcare NHS Trust, London, United Kingdom	Hospital Prof. Dr. Fernando Fonseca, Lisbon, Portugal	General Hospital, Vienna, Austria
**Hospital characteristics**	University hospital<br>60 paediatric beds	Teaching hospital<br>59 paediatric beds	University hospital<br>46 paediatric beds	Community hospital<br>91 paediatric beds	University hospital<br>134 paediatric beds
**Emergency department characteristics**[a]	6,500 children/year<br><br>Urban<br><br>Mixed high and low socio-economic status	9,500 children/year<br><br>Urban<br><br>Generally low socio-economic status	27,000 children/year<br><br>Urban<br><br>Mixed high and low socio-economic status	60,000 children/year<br><br>Mixed urban/rural<br>Generally low socio-economic status	22,000 children/year<br><br>Urban<br><br>Mixed high and low socio-economic status
**Number of patients included**	18,590	10,583	15,556	53,175	21,300
**Inclusion period**	01-01-2012 to 31-12-2014	01-05-2014 to 31-10-2015	01-07-2014 to 28-02-2015	01-03-2014 to 28-02-2015	01-01-2014 to 31-12-2014
**Number of patients during:**					
**- Office hours**	8,915 (48.0%)	4,229 (40.0%)	5,635 (36.2%)	22,470 (42.3%)	9,168 (43.0%)
**- Out-of-office hours**	9,675 (52.0%)	6,354 (60.0%)	9,921 (63.8%)	30,705 (57.7%)	12,132 (57.0%)
**Median age,** ***yrs (IQR[b])***	4.3 (1.4 – 9.8)	5.7 (1.9 – 11.6)	3.9 (1.5 – 8.8)	4.7 (1.9 – 9.5)	3.9 (1.6 – 8.3)
**MTS,** ***n (%)***					
- **High urgent**	2,427 (13.1)	1,515 (14.3)	1,605 (10.3)	6,222 (11.7)	1,084 (5.0)
- **Urgent**	8,744 (47.0)	5,110 (48.3)	3,961 (25.5)	10,951 (20.6)	3851 (18.1)
- **Low urgent**	6,849 (36.8)	3,857 (36.4)	9,990 (64.2)	36,002 (67.7)	15,314 (71.9)
- **Missing**	570 (3.1)	101 (0.1)	0 (0.0)	0 (0.0)	1,051 (4.9)
**Presenting problem,** ***n (%)***					
- **Medical**	12.206 (65.7)	5,163 (48.8)	11.520 (74.1)	43,814 (82.4)	19,174 (90.0)
- **Trauma**	5,814 (31.3)	5,321 (50.3)	4,036 (25.9)	9,361 (17.6)	1,034 (4.9)
- **Missing**	570 (3.1)	99 (0.9)	0 (0.0)	0 (0.0)	1,092 (5.1)

[a]All emergency departments (ED) are paediatric only, except for the Maasstad Hospital, which is mixed adult-paediatric. The Erasmus MC-Sophia Children's hospital has a mixed adult-paediatric ED since October 2014 (3 months in total during the inclusion period).
[b]IQR = Interquartile range.

Confidential: For Review Only

Figure 1. Presenting problems

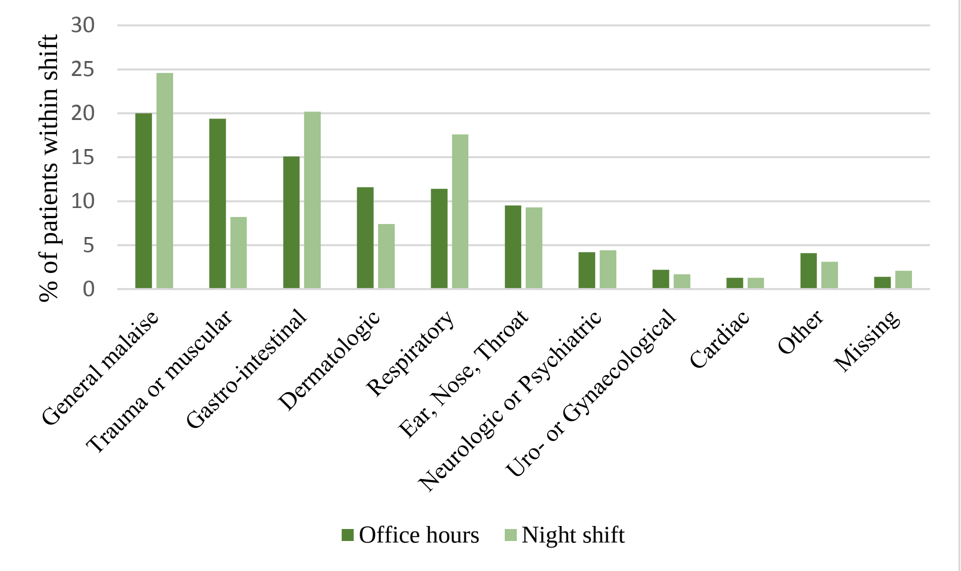

Confidential: For Review Only

Supplementary files

Supplementary table.1 Immediate life-saving interventions[a]
1. Airway and breathing support, including intubation or emergent non-invasive positive pressure ventilation.
2. Electrical therapy, including defibrillation, emergent cardioversion, or external pacing.
3. Procedures, including chest needle decompression, pericardiocentesis, or open thoracotomy.
4. Hemodynamic support, including significant intravenous fluid resuscitation in the setting of hypotension, blood administration, or control of major bleeding.
5. Emergency medications, including naloxone, dextrose, atropine, adenosine, epinephrine, or vasopressors

[a]Adapted from Lee et. al.(24)

Supplementary table 2. Presenting problems definitions

Subgroups[a]	MTS Flowchart
**General malaise**	Crying baby. Unwell child. Worried parent. Irritable child. Unwell adult.
**Trauma or muscular**	Assault. Falls. Head injury. Limb problems. Major trauma. Torso injury. Neck pain. Back pain. Limping child.
**Gastro-intestinal**	Abdominal pain in adults. Abdominal pain in children. Diarrhoea and vomiting. GI Bleeding.
**Respiratory**	Asthma. Shortness of breath in adults. Shortness of breath in children.
**Eear, Nose, Throat**	Ear problems. Sore throat. Facial problems.
**Neurologic or Psychiatric**	Behaving strangely. Fits. Headache. Apparently drunk. Collapsed adult. Overdose and poisoning. Self-harm. Mental illness.
**Dermatologic**	Bites and stings. Burns and scalds. Wounds. Abscesses and local infections. Rashes.
**Uro- or Gynaecological**	Testicular pain. Urinary problems. Sexually acquired infection. Pregnancy. PV Bleeding.
**Cardiac**	Chest pain. Palpitations.
**Other**	Allergy. Dental problems. Diabetes. Exposure to chemicals. Eye problems. Foreign body. Major incidents – primary. Major incidents – secondary. General/other.

[a]Subgroups of presenting problems (MTS Flowchart), based on a previous study (23)

Confidential: For Review Only

Supplementary table 3. Presenting problems

N (%)	Office hours (n=50,417)	Evening shift (n=36,429)	Night shift (n=14,138)	Day shift weekend (n=18,220)
**General malaise**	10,069 (20.0)	7,021 (19.3)	3,473 (24.6)	3,784 (20.8)
**Trauma or muscular**	9,785 (19.4)	7,281 (20.0)	1,161 (8.2)	3,111 (17.1)
**Gastro-intestinal**	7,613 (15.1)	5,274 (14.5)	2,860 (20.2)	2,470 (13.6)
**Dermatologic**	5,864 (11.6)	4,851 (13.3)	1,044 (7.4)	2,864 (15.7)
**Respiratory**	5,686 (11.3)	3,833 (10.5)	2,494 (17.6)	1,947 (10.7)
**Ear, Nose, Throat**	4,777 (9.5)	3,596 (9.9)	1,320 (9.3)	1,870 (10.3)
**Neurologic or Psychiatric**	2,115 (4.2)	1,384 (3.8)	628 (4.4)	566 (3.1)
**Uro- or Gynaecological**	1,126 (2.2)	800 (2.2)	240 (1.7)	416 (2.3)
**Cardiac**	635 (1.3)	438 (1.2)	177 (1.3)	148 (0.8)
**Other**	2,084 (4.1)	1,489 (4.1)	443 (3.1)	733 (4.0)
**Missing**	690 (1.4)	462 (1.3)	298 (2.1)	311 (1.7)

Confidential: For Review Only

Supplementary table 4. Management adjusted regression analyses, per hospital					
	**Erasmus MC - Sophia Children's Hospital, Rotterdam the Netherlands**	**Maasstad Hospital, Rotterdam, the Netherlands**	**St. Mary's hospital Imperial College Healthcare NHS Trust, London, United Kingdom**	**Hospital Prof. Dr. Fernando Fonseca, Lisbon, Portugal**	**General Hospital, Vienna, Austria**
Total n=119,204 aOR[a] (95% CI)	n = 18,590	n = 10,583	n = 15,556	n = 53,175	n = 21,300
**Lab**					
- Evening shift	0.75 (0.69 – 0.82) *	0.82 (0.71 – 0.94) *	0.73 (0.63 – 0.83)*	1.00 (0.94 – 1.07)	0.61 (0.57 – 0.66) *
- Night shift	0.77 (0.68 – 0.87) *	0.86 (0.71 – 1.04)	0.71 (0.60 – 0.84) *	1.03 (0.95 – 1.11)	0.68 (0.61 – 0.77) *
- Day shift weekend	0.90 (0.81 – 0.99)*	0.85 (0.71 – 1.02)	0.68 (0.57 – 0.81)*	1.15 (1.07 – 1.25) *	0.82 (0.74 – 0.90) *
**Imaging**					
- Evening shift	0.73 (0.67 – 0.80) *	2.18 (1.93 – 2.47) *	0.77 (0.68 – 0.87) *	0.94 (0.88 – 0.99) *	0.59 (0.52 – 0.68) *
- Night shift	0.53 (0.46 – 0.62)*	1.05 0.84 – 1.30	0.52 (0.43 – 0.62) *	0.68 0.63 – 0.74 *	0.55 (0.44 – 0.67)*
- Day shift weekend	0.85 (0.76 – 0.94)	2.00 (1.72 – 2.34) *	0.81 (0.69 – 0.94) *	0.94 (0.88 – 1.02)	1.00 (0.86 – 1.16)
**Treatment**					
- Evening shift	1.13 (1.04 – 1.22) *	0.78 0.68 – 0.89) *	1.35 (1.25 – 1.47)*	0.99 (0.93 – 1.04)	1.44 (1.32 – 1.58) *
- Night shift	1.32 (1.17 – 1.50)*	1.03 (0.86 – 1.24)	1.55 (1.40 – 1.72) *	1.53 (1.43 – 1.64) *	2.32 (2.06 – 2.62) *
- Day shift weekend	1.12 1.01 – 1.23*	0.89 (0.75 – 1.06)	1.31 (1.19 – 1.54) *	1.12 (1.03 – 1.21) *	1.38 (1.23 – 1.55) *
**Hospital admission**					
- Evening shift	1.20 (1.09 – 1.31) *	1.05 (0.93 – 1.18)	0.92 (0.80 – 1.06)	1.05 (0.95 – 1.16)	0.93 (0.80 – 1.09)
- Night shift	1.43 (1.25 – 1.63) *	1.44 (1.22 – 1.71) *	0.95 (0.80 – 1.19)	1.51 (1.35 – 1.69) *	1.06 (0.86 – 1.30)
- Day shift weekend	1.15 (1.03 – 1.29 *	1.04 (0.89 – 1.21)	0.71 (0.59 – 0.85)*	1.09 (0.95 – 1.24)	1.32 (1.10 – 1.58) *

[a]aOR = Adjusted Odds Ratio. Adjusted for age, gender, urgency level, abnormal vital signs, presenting problem
*P-value ≤0.05. If not stated: p-value >0.05

Confidential: For Review Only

Supplementary figure 1. Missing vital signs[a]

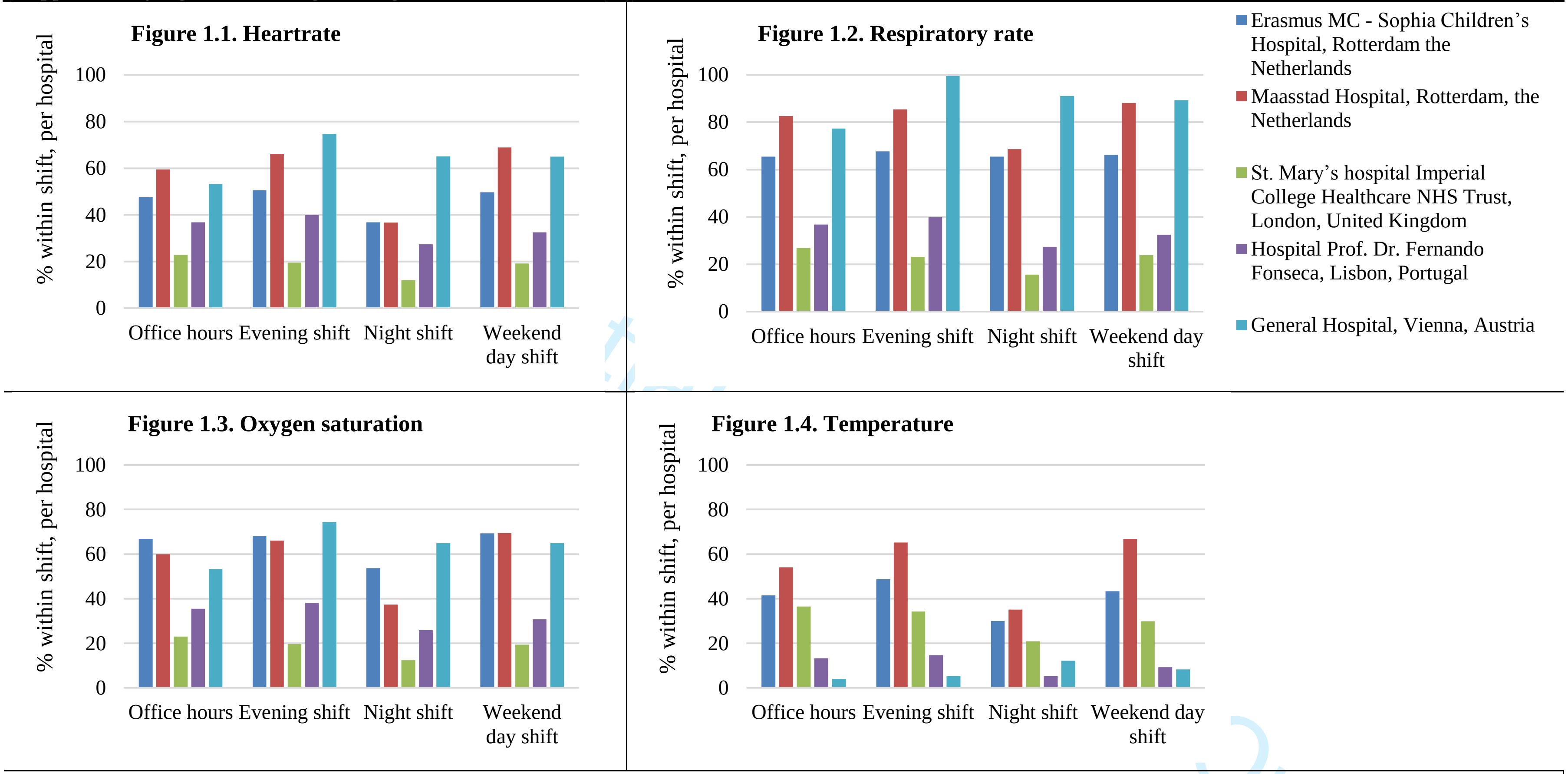

[a]Missing vital signs were imputed using a multiple imputation model including age, vital signs, MTS urgency, presenting problem and disposition after the ED. This imputation process resulted in twenty-five datasets on which statistical analyses were performed and pooled for a final result.(25) Imputation was performed by using the MICE imputation package in R, version 2.15.2.

BMJ Paediatrics Open

Management of children visiting the emergency department during out-of-office hours: an observational study

Journal:	*BMJ Paediatrics Open*
Manuscript ID	bmjpo-2020-000687.R2
Article Type:	Original research
Date Submitted by the Author:	11-Jun-2020
Complete List of Authors:	Schinkelshoek, Gina; Erasmus MC Sophia Children Hospital<br>Borensztajn, Dorine; Erasmus MC Sophia Children Hospital, Department of Pediatrics<br>Zachariasse, Joany; Erasmus MC Sophia Children Hospital, Department of Pediatrics<br>Maconochie, Ian; Imperial College Healthcare NHS Trust, Paediatric Emergency Department<br>Alves, Claudio; Professor Doctor Fernando Fonseca Hospital, General Paediatrics<br>Freitas , Paulo ; Professor Doctor Fernando Fonseca Hospital, Intensive Care Unit<br>Smit, Frank; Maasstad Hospital, Paediatrics<br>van der Lei, Johan; Erasmus Medical Center, Department of Medical Informatics<br>Steyerberg, Ewout; Erasmus Medical Center, Public Health<br>Greber-Platzer, Susanne; Medical University of Vienna, Division of Pediatric Pulmology, Allergology and Endocrinology, Department of Pediatrics and Adolescent Medicine<br>moll, Henriette; Erasmus MC Sophia Children Hospital, Department of Pediatrics
Keywords:	Accident & Emergency, General Paediatrics, Paediatric Practice

SCHOLARONE™
Manuscripts

I, the Submitting Author has the right to grant and does grant on behalf of all authors of the Work (as defined in the below author licence), an exclusive licence and/or a non-exclusive licence for contributions from authors who are: i) UK Crown employees; ii) where BMJ has agreed a CC-BY licence shall apply, and/or iii) in accordance with the terms applicable for US Federal Government officers or employees acting as part of their official duties; on a worldwide, perpetual, irrevocable, royalty-free basis to BMJ Publishing Group Ltd ("BMJ") its licensees and where the relevant Journal is co-owned by BMJ to the co-owners of the Journal, to publish the Work in this journal and any other BMJ products and to exploit all rights, as set out in our licence.

The Submitting Author accepts and understands that any supply made under these terms is made by BMJ to the Submitting Author unless you are acting as an employee on behalf of your employer or a postgraduate student of an affiliated institution which is paying any applicable article publishing charge ("APC") for Open Access articles. Where the Submitting Author wishes to make the Work available on an Open Access basis (and intends to pay the relevant APC), the terms of reuse of such Open Access shall be governed by a Creative Commons licence – details of these licences and which Creative Commons licence will apply to this Work are set out in our licence referred to above.

Other than as permitted in any relevant BMJ Author's Self Archiving Policies, I confirm this Work has not been accepted for publication elsewhere, is not being considered for publication elsewhere and does not duplicate material already published. I confirm all authors consent to publication of this Work and authorise the granting of this licence.

Confidential: For Review Only

MANAGEMENT OF CHILDREN VISITING THE EMERGENCY DEPARTMENT DURING OUT-OF-OFFICE HOURS: AN OBSERVATIONAL STUDY

Gina Schinkelshoek[1*], Dorine M. Borensztajn[1*], Joany M. Zachariasse[1], Ian K. Maconochie[2], Claudio F. Alves[3], Paulo Freitas[4], Frank J. Smit[5], Johan van der Lei[6], Ewout W. Steyerberg[7], Susanne Greber-Platzer[8], Henriëtte A. Moll[1]

Affliations:
[1]Erasmus MC – Sophia Children's Hospital, Department of General Paediatrics, Rotterdam, The Netherlands.
[2]Imperial College NHS Healthcare Trust, Department of Paediatric Accident and Emergency, London, United Kingdom.
[3]Hospital Prof. Dr. Fernando Fonseca, Department of Paediatrics, Lisbon, Portugal.
[4]Hospital Prof. Dr. Fernando Fonseca, Intensive Care Unit, Lisbon, Portugal.
[5]Maasstad Hospital, Department of Paediatrics, Rotterdam, the Netherlands.
[6]Erasmus MC- University Medical Centre Rotterdam, Department of Medical Informatics, Rotterdam, The Netherlands.
[7]Leiden University Medical Centre, Department of Biomedical Data Sciences, Leiden, The Netherlands.
[8]Medical University Vienna, Department of Paediatrics and Adolescent Medicine, Vienna, Austria.
*Gina Schinkelshoek and Dorine M. Borensztajn contributed equally to this paper.

Corresponding Author:
Prof. Dr. H.A. Moll
Department of General Paediatrics
Erasmus MC – Sophia Children's Hospital
P.O. Box 2060, 3000 CB, Rotterdam, The Netherlands.
Telephone: +31 10 703 61 96
Fax: +31 10 703 66 85
Email: H.A.Moll@erasmusmc.nl

Word count:
2778 words

Confidential: For Review Only

What is already known on this topic:

Concerns exist that emergency care may differ during out-of-office hours and office hours. There has been growing attention for paediatric emergency care at the emergency department recently. However, little is known about children visiting the emergency department during out-of-office hours.

What this study adds:

Children visiting the emergency department during the night were more seriously ill than during office hours. Fewer diagnostic tests, more treatment and more hospital admissions were reported during the night, which could not be explained by correcting for illness severity.

Confidential: For Review Only

ABSTRACT

Background
The aim was to study the characteristics and management of children visiting the Emergency Department (ED) during out-of-office hours.
Methods
We analysed electronic health record data from 119,204 children visiting one of five EDs in four European countries. Patient characteristics and management (diagnostic tests, treatment, hospital admission and Paediatric Intensive Care Unit admission) were compared between children visiting during office hours and evening shifts, night shifts and weekend day shifts. Analyses were corrected for age, gender, MTS urgency, abnormal vital signs, presenting problem and hospital.
Results
Patients presenting at night were younger (median (IQR) age: 3.7 (1.4 – 8.2) years versus 4.8 (1.8 – 9.9)), more often classified as high urgent (16.3% versus 9.9%) and more often had ≥ 2 abnormal vital signs (22.8% versus 18.1%), compared to office hours. After correcting for disease severity, laboratory and radiological tests were less likely to be requested (aOR: 0.82, 95% CI: 0.78 – 0.86 and aOR: 0.64, 95% CI: 0.60 – 0.67 respectively); treatment was more likely to be undertaken (aOR: 1.56, 95% CI: 1.49 – 1.63) and patients were more likely to be admitted to the hospital (aOR: 1.32, 95% CI: 1.24 -1.41) at night. Patterns in management during out-of-office hours were comparable between the different hospitals, with variability remaining.
Conclusions
Children visiting during the night are relatively more seriously ill, highlighting the need to keep improving emergency care on a 24 hours a day basis. Further research is needed to explain the differences in management during the night and how these differences affect patient outcome.

Confidential: For Review Only

INTRODUCTION

Concerns exist that emergency care may differ during out-of-office hours, i.e. evening, night and/or weekend shifts, compared to office hours. For example, higher mortality rates were found during weekend visits in adult patients with sepsis presenting to the Emergency Department (ED).(1) Some suggest that worse patient outcome during out-of-office hours can, partly, be explained by higher illness severity and not by time of arrival as independent factor.(2, 3) When looking at the paediatric population, higher mortality rates were observed among children admitted to the PICU during out-of-office hours.(4) However, other studies analysing PICU patients are in conflict with this finding (5-9).

Over the past few years, increasing attention has been paid to paediatric emergency care, leading to different guidelines and standards for children visiting the ED.(10-14) These guidelines aim to achieve optimal utilization of available resources, optimize patient outcome and thereby improve quality of care. A recent study reported that main standards of care are fulfilled in European EDs, but some areas of improvement still exist.(15)

However, little research has focused specifically on children visiting the ED during out-of-office hours. Recently, variation in paediatric ED utilization patterns and the presentation of these children by shift of arrival has been reported.(16) But this study was a single centre study, which may limit the generalizability. Moreover, no study has analysed resource utilization at paediatric EDs during out-of-office hours, except for studies analysing children with a specific presenting problem.(17-19)

It is important to obtain insight into the characteristics and management of the whole paediatric patient population attending the ED with its variable presenting problems, as this may be helpful in optimizing guidelines and resource allocation, and thereby in improving the quality of care on a 24 hours a day basis.

Confidential: For Review Only

The aim of this study was to compare patient characteristics and management of paediatric patients visiting the ED during out-of-office hours to office hours, in four different European countries.

Confidential: For Review Only

METHODS

Study design, setting, patients

This study is part of the TrIAGE project (Triage Improvement Across General Emergency departments for paediatric patients), a prospective observational study.(20) This project captured routinely collected, standardized, electronic health record data of all non-scheduled ED visits of children <16 years in five different hospitals in four different countries. The five participating hospitals, selected by convenience sampling, were: Erasmus Medical Centre, the Netherlands; Maasstad Hospital, the Netherlands; St. Mary's hospital Imperial College Healthcare NHS Trust, United Kingdom; Hospital Prof. Dr. Fernando Fonseca, Portugal; Vienna General Hospital, Austria. The enrolment period varied from 8 to 36 months between 2012 and 2015.

At the beginning of the study, a minimum dataset of variables for collection was defined. If necessary, entries in the electronic health data were added or modified. Nurses at the ED were informed about the study and encouraged to fill in the entire medical records, including vital signs, which is in principle routine data. During the study this routinely collected data was documented in the electronic, medical records by nurses at the ED. Data completeness and accuracy of the data was reviewed using a checklist of quality control. Sometimes, data was completed by information or vital signs from medical records documented by physicians at the ED. Coded data were extracted from the electronic hospital information systems and thereafter transferred to the principal investigator as an encrypted file. Careful data harmonization and quality checks were conducted. The requirement for informed consent was obtained by all hospitals and the study was approved by the medical ethical committees of all participating institutions.

Confidential: For Review Only

Patient and Public Involvement statement

Patients or the public were not involved in this research.

Definitions

Office hours versus out-of-office hours

Office hours were defined as Monday until Friday, between 08:00 am and 05:59 pm, also referred to as day shift week. We compared office hours, as reference shift, to out-of-office hours, divided into evening shift, night shifts and day shifts during the weekend. Evening shifts were defined as Monday until Sunday between 6:00 pm and 10:59 pm and night shifts as Monday until Sunday between 11:00 pm and 07:59 am, based on previous literature. (5, 9, 17) Weekend day shifts were defined as Saturday and Sunday between 08:00 am and 5:59 pm.

Patient characteristics

For this study, the following data were extracted from the database: age, gender, time and date of arrival, Manchester Triage System (MTS) urgency, vital signs and presenting symptom (based on the MTS flowchart). The MTS urgency consists of five categories: emergent, very urgent, urgent, standard and non-urgent.(21-23) These categories were divided in three groups: high urgent (emergent or very urgent, <10 minutes waiting time), urgent (urgent, <60 minutes waiting time), low urgent (standard or non-urgent, ≥ 60 minutes waiting time) . Vital signs included heart rate, respiratory rate, oxygen saturation and temperature. Vital signs were considered abnormal based on the Advanced Paediatric Life Support (APLS) reference values, with fever defined as temperature ≥38.5ºC.(24) The MTS flowchart was used to create subgroups of presenting problems. These subgroups, based on a previous study (25), included medical (respiratory, ear, nose and throat (ENT) problems, gastro-intestinal problems,

https://mc.manuscriptcentral.com/bmjpo

Confidential: For Review Only

neurological or psychiatric problems, general malaise, uro- or gynaecological problems, dermatologic, others) and trauma (trauma or muscular and wounds). (supplementary table 1.1)

Management

Management was defined as diagnostics, (laboratory tests and imaging), treatment at the ED and hospital admission directly after ED visit. Laboratory tests were divided into: blood tests, including cultures; and urine tests, including cultures. Imaging was divided into: x-ray, ultrasound and Computed Tomography (CT).

Treatment was divided into: oral medication, inhalation medication, intravenous (IV) medication or IV fluids and Immediate Lifesaving Interventions (ILI) (supplementary table 2) (26).

In the multivariable regression models, we defined laboratory diagnostics as any form of laboratory tests, radiological diagnostics as any form of imaging and treatment as any form of treatment.

Hospital admission included patients who were admitted to the general ward or to the PICU. PICU admission was defined as admission to the PICU directly from the ED.

Statistical analysis

In descriptive statistics of patient characteristics continuous, skewed variables are shown as median with interquartile range (IQR). Nominal and ordinal variables are shown as number of patients with percentage within shift. Nominal and ordinal variables considering management are shown as percentages within shift, with inter-hospital ranges.

In multivariable regression analyses differences in management were compared between office hours and evening, night and weekend day shifts. To study the independent role of shift of arrival, the regression analyses were adjusted for age, gender, MTS urgency, abnormal vital

Confidential: For Review Only

signs, presenting problem and hospital. We performed stratified analyses, by conducting the adjusted regression analyses for each hospital separately, to provide insight into inter-hospital differences.

Missing data for vital signs were imputed using a multiple imputation model. (table 1) These missing data were assumed to be conditional on other variables included in the database, i.e. missing at random. (supplement 3) Sensitivity analyses were performed by analysing a database restricted to complete cases. (supplement 4) (27, 28)

Results were considered statistically significant at a P-value of ≤0.05.

Initial analyses were performed with SPSS software, version 21 (IBM SPSS statistics, IBM corporation, Armonk, NY).

Confidential: For Review Only

RESULTS

Patient population

In total, 119,209 patients, ≤16 years, visited the participating EDs. Five patients were excluded owing to missing arrival time or date, leaving 119,204 patients for analysis, with 50,417 (42.3%) of these patients who visited the ED during office hours versus 68,787 (57.7%) during out-of-office hours. Of those, 18,220 (15.3%) visited during weekend day shifts, 36,429 (30.6%) during the evening and 14,138 (11.9%) during the night.

Patients who visited the ED during the night were younger, were more often classified by MTS as high urgent, and more often had two or more abnormal vital signs (table 2).

During office hours, general malaise (20.0%), trauma or muscular (19.4%) and gastro-intestinal problems (15.1%) were the most common presenting problems (figure 1). During the night, relatively more general malaise (24.6%), gastro-intestinal problems (20.2%), respiratory (17.6%) and fewer trauma cases (8.2%) were observed. Presenting problems during the evening and weekend day shifts were similar compared to office hours. (supplementary table 1.2)

Management

During out-of-office hours, fewer diagnostic tests, more treatment and more hospital and PICU admissions were conducted. (table 3)

During the evening and weekend day shift, fewer urine and blood tests were performed.

During the evening, night and weekend day shifts, imaging was performed less often, except for X-ray during the evening shift. Treatment with oral, inhaled and intravenous medication were given more often, especially during the night. Also, during the night, slightly more immediate lifesaving interventions were seen, whilst more hospital and ICU admissions were observed.

Confidential: For Review Only

In multivariable regression analyses, the results were adjusted for age, gender, urgency, abnormal vital signs and presenting problem and hospital. (table 4)

Laboratory and imaging diagnostics were significantly less likely to be performed during the evening and night shifts. Also, patients were significantly more likely to receive treatment during all out-of-office hours shifts and more likely to be admitted to the hospital during the night shift. After adjusting, no association was found between PICU admission and out-of-office hours visits.

When analysing a database consisting only of complete cases for vital signs, overall results were comparable to the analyses in the imputation database. (supplement 4)

Inter-hospital analyses

Hospital characteristics are shown in table 5 and supplementary table 5.1-5.5. Some inter-hospital variation in management was observed. (table 2) The multivariable regression analyses, adjusted for age, gender, urgency level, abnormal vital signs and presenting problem, were performed for each hospital separately in stratified analyses. (supplementary table 6) Overall patterns in management during out-of-office hours were comparable between the different hospitals, with some variation remaining. For example, at two hospitals no significant difference was found in hospital admissions during the night. At another hospital imaging was requested more frequently during evening and weekend shifts.

Confidential: For Review Only

DISCUSSION

In this large observational study of 119,204 visits in five EDs in Europe, we demonstrate differences in acuity level and management of children visiting the ED during out-of-office hours compared to office hours.

We found that children visiting during the night were relatively more seriously ill. Secondly, after correcting for patient characteristics, illness severity and hospital, we observed fewer laboratory and radiological tests whilst children were more likely to receive treatment at the ED and to be admitted to the hospital during especially the night. No association was found between out-of-office hours visits and PICU admission, after statistically correcting for disease severity. Overall, the trends in out-of-office hours care were comparable between the different hospitals, with variability remaining.

In a recent study, ED utilization patterns of paediatric patients through the day was analysed.(16) The distribution of visits over the evening and night shifts was quite similar to our results. Also, in line with our results, they observed that children during the night are relatively more ill. So, although the proportion of patients visiting during the night is the smallest, the acuity of this group is the highest, which shows the importance of the same quality of care on a 24 hours a day basis. In contrast to our results, no association between shift of arrival and hospital admission was reported. However, this was a single centre study, which limits the generalizability.

Variation in management during out-of-office hours, most pronounced during the night, could not be explained by illness severity. Reducing variation in care could result in lower resource utilization and costs, and could improve the use of time, space and staff. (29) To obtain more standardized care, also during out-of-office hours, it is essential to understand which factors contribute to the differences we observed.

https://mc.manuscriptcentral.com/bmjpo

Confidential: For Review Only

At first, different levels of staff during the night might influence management. Several studies found differences in resource use (30-34) and admission rates (35) depending on physician background and experience.

Secondly, availability of resources during night shifts, such as imaging resources or the availability of specialized radiologists to assess these results during the night, might contribute to the lower use of imaging during the night. (36)

Our results raise the question whether these higher treatment and admission rates, and the lower use of diagnostic imaging during the night, might have been unwarranted and contribute to unnecessary costs. Earlier research reported that higher resource utilization is related to higher costs, but not to lower admission rates. (37-39) One of the hospitals in our study showed no difference in diagnostics during the night, in contrast to the other hospitals, but still showed higher admission rates compared to office hours.

We speculate that our results might also partly be explained by a more pragmatic approach of the patient during the night, resulting in fewer diagnostic tests, more treatment and more hospital admissions. Previous literature did report higher rates of antibiotic prescriptions (17) and recommendations to admit to the hospital (40), in children visiting the ED during the night. Additionally, parental demand might contribute to these results. (41) Furthermore, in contrast to general hospital admission, the higher rates of PICU admission during the night, could be explained by illness severity and case mix. The indications for PICU admissions are more standardized compared to indications for general hospital and therefore less influenced by non-patient factors.

These results point to the need for further research to explain the differences in clinical decisions during the night, by assessing differences in standards of care during out-of-office hours, such as availability of experienced staff and availability of diagnostics and equipment.(14) Moreover, it is important to obtain insight in how these differences in

https://mc.manuscriptcentral.com/bmjpo

Confidential: For Review Only

management affect patient outcome, for example rehospitalisation, morbidity and 30-day mortality. Implementing guidelines for management of children with specific conditions, could help to reduce variation in management within and between hospitals, which optimize resource allocation and costs. Moreover, these guidelines could allow for better comparisons between the hospitals in further research.

Other studies on the topic of out-of-office hours care, including adult patients at the ED and paediatric patients at the PICU, described worse patient outcome during the night and evening, represented by higher mortality rates. (1, 4, 42) We did not use mortality as the main outcome as the mortality in our dataset was very low (16 patients, 0.01%), which is in line with previous literature. (43, 44)

Strengths and limitations

A major strength of this study is the large number of patients from different centres, including both university as teaching hospitals. This increases the generalizability of the results. Moreover, we adjusted for multiple variables concerning illness severity to analyse the independent role of time of visit to management.

To appreciate the results, some limitations have to be considered. Hospitals were selected by convenience sampling, which influences the generalizability of the study. The enrolment period varied between the different hospitals. However, each hospital was followed for at least 8 months and in all hospitals a winter season was included to increase the comparability of the periods. Also, to our knowledge, there were no major changes or differences in for example vaccination status, occurrence of epidemics or other incidents during the total enrolment period (2012-2015).

Secondly, the analyses were based on routinely collected data. This results in some missing data, which we dealt with using multiple imputation for vital signs. The proportion of missing

Confidential: For Review Only

data for vital signs was comparable with previous studies (45-47) and varied between the hospitals, which can be expected due to patient mix and differences in local policies.

Moreover, it is possible that there are more variables representing illness severity and influencing management, that were not included. These factors can be divided into patient- and hospital-related factors. Patient-related factors are e.g. comorbidity, socio-economic status or ethnic background. When looking at hospital-related factors, (experience level of) staff, resource availability and bed availability could possibly contribute to the differences in management. To deal with hospital-related differences, we adjusted our regression analyses for hospital. Furthermore, we performed the regression analyses for each hospital separately. These stratified analyses demonstrated that overall results were comparable between the hospitals, but some variation still remained. However, we did not have the specific information about the aforementioned hospital-level factors for each individual visit, to include in the analyses. It would be interesting to analyse the potential effects of these factors in future research.

Conclusion

Children visiting during the night are relatively more seriously ill than those visiting during office hours. This highlights the need to keep improving emergency care on a 24 hours a day basis. Furthermore, differences in management are present depending on the time of ED visit. Especially during the night, diagnostic tests are performed less frequently, whilst treatment is initiated more often and more children are admitted to the hospital than during office hours. These patterns are not explained by illness severity. Further research should focus on exploring the reasons for these differences and how these differences affect patient outcome.

Confidential: For Review Only

FUNDING STATEMENT

This research received no specific grant from any funding agency in the public, commercial or not-for-profit sectors.

COMPETING INTERESTS

All authors have no conflicts of interest relevant to this article to disclose.

CONTRIBUTORSHIP STATEMENT

Gina Schinkelshoek: Conceptualization, data curation, formal analysis, investigation, writing original draft, review and editing.

Dorine M. Borensztajn: Conceptualization, data curation, formal analysis, investigation, writing original draft, review and editing.

Joany M. Zachariasse: Data curation, formal analysis, investigation, review and editing.

Susanne Greber-Platzer: Data curation, investigation, review and editing.

Claudio F. Alves: Data curation, investigation, review and editing.

Paulo Freitas: Data curation, investigation, review and editing.

Frank J. Smit: Data curation, investigation, review and editing.

Ian K. Maconochie: Data curation, investigation, review and editing.

Johan van der Lei: Conceptualization, data curation, review and editing.

Ewout W. Steyerberg: Data curation, review and editing.

Henriëtte A. Moll: Conceptualization, data curation, investigation, project administration, supervision, review and editing.

All authors approved the final manuscript as submitted and agree to be accountable for all aspects of work.

Confidential: For Review Only

DATA SHARING STATEMENT

Data from this study are available upon request to the corresponding author of the study (h.a.moll@erasmusmc.nl), subject to local rules and regulations.

Confidential: For Review Only

References

1. Powell ES, Khare RK, Courtney DM, Feinglass J. The weekend effect for patients with sepsis presenting to the emergency department. J Emerg Med. 2013 Nov;45(5):641-8.
2. Mikulich O, Callaly E, Bennett K, O'Riordan D, Silke B. The increased mortality associated with a weekend emergency admission is due to increased illness severity and altered case-mix. Acute Med. 2011;10(4):182-7.
3. Walker AS, Mason A, Quan TP, Fawcett NJ, Watkinson P, Llewelyn M, et al. Mortality risks associated with emergency admissions during weekends and public holidays: an analysis of electronic health records. Lancet. 2017 May 09.
4. Arias Y, Taylor DS, Marcin JP. Association between evening admissions and higher mortality rates in the pediatric intensive care unit. Pediatrics. 2004 Jun;113(6):e530-4.
5. Peeters B, Jansen NJ, Bollen CW, van Vught AJ, van der Heide D, Albers MJ. Off-hours admission and mortality in two pediatric intensive care units without 24-h in-house senior staff attendance. Intensive Care Med. 2010 Nov;36(11):1923-7.
6. McShane P, Draper ES, McKinney PA, McFadzean J, Parslow RC, Paediatric Intensive Care Audit N. Effects of out-of-hours and winter admissions and number of patients per unit on mortality in pediatric intensive care. J Pediatr. 2013 Oct;163(4):1039-44 e5.
7. Thabet FC, alHaffaf FA, Bougmiza IM, Bafaqih HA, Chehab MS, alMohaimeed SA. Off-Hours Admissions and Mortality in PICU Without 24-Hour Onsite Intensivist Coverage. J Intensive Care Med. 2018 Jan 1:885066618778824.
8. Hixson ED, Davis S, Morris S, Harrison AM. Do weekends or evenings matter in a pediatric intensive care unit? Pediatr Crit Care Med. 2005 Sep;6(5):523-30.
9. Numa A, Williams G, Awad J, Duffy B. After-hours admissions are not associated with increased risk-adjusted mortality in pediatric intensive care. Intensive Care Med. 2008 Jan;34(1):148-51.
10. American Academy of Pediatrics Committee on Pediatric Emergency M, American College of Emergency Physicians Pediatric C, Emergency Nurses Association Pediatric C. Joint policy statement--guidelines for care of children in the emergency department. Ann Emerg Med. 2009 Oct;54(4):543-52.
11. Gausche-Hill M, Wiebe RA. Guidelines for preparedness of emergency departments that care for children: A call to action. Annals of Emergency Medicine. [doi: 10.1067/mem.2001.114069]. 2001;37(4):389-91.
12. Remick K, Gausche-Hill M, Joseph MM, Brown K, Snow SK, Wright JL, et al. Pediatric Readiness in the Emergency Department. Pediatrics. 2018 Nov;142(5).
13. Facing the Future: Standards for children in emergency care settings. Royal College of Paediatrics and Child Health; 2018 [cited 2019 October]; Available from: https://www.rcpch.ac.uk/sites/default/files/2018-06/FTFEC%20Digital%20updated%20final.pdf.
14. Standards of Care for Children in Emergency Departments (Third version). International Federation for Emergency Medicine; 2012 [updated April 2019; cited 2019 October]; Available from: https://www.ifem.cc/wp-content/uploads/2019/06/Standards-of-Care-for-Children-in-Emergency-Departments-V3-2019.pdf.
15. Mintegi S, Maconochie IK, Waisman Y, Titomanlio L, Benito J, Laribi S, et al. Pediatric Preparedness of European Emergency Departments: A Multicenter International Survey. Pediatr Emerg Care. 2020 May 8.
16. Almubarak H, Meckler G, Doan Q. Factors and outcomes associated with paediatric emergency department arrival patterns through the day. Paediatr Child Health. 2019 Aug;24(5):323-9.

Confidential: For Review Only

17. Roggen I, van Berlaer G, Gordts F, Pierard D, Hubloue I. Acute sore throat in children at the emergency department: best medical practice? Eur J Emerg Med. 2015 Oct;22(5):343-7.
18. Johnson EK, Graham DA, Chow JS, Nelson CP. Nationwide emergency department imaging practices for pediatric urolithiasis: room for improvement. J Urol. 2014 Jul;192(1):200-6.
19. Daverio M, Bressan S, Gregori D, Babl FE, Mackay MT. Patient and Process Factors Associated With Type of First Neuroimaging and Delayed Diagnosis in Childhood Arterial Ischemic Stroke. Acad Emerg Med. 2016 Sep;23(9):1040-7.
20. Zachariasse JM, Nieboer D, Maconochie IK, Smit FJ, Alves CF, Greber-Platzer S, et al. Development and validation of a Paediatric Early Warning Score for use in the emergency department: a multicentre study. Accepted for publication in Lancet Child and Adolescent Health. 2020.
21. De Caluwé R, Manchester Triage G. Triage voor de spoedeisende hulp: Elsevier Gezondheidszorg; 2007.
22. van Veen M, Steyerberg EW, van't Klooster M, Ruige M, van Meurs AHJ, van der Lei J, et al. The Manchester triage system: improvements for paediatric emergency care. Emergency Medicine Journal. 2012;29(8):654-9.
23. Mackway-Jones K, Marsden J, Windle J. Emergency Triage: Manchester Triage Group. Third ed. Oxford, United Kingdom: John Wiley & Sons; 2013.
24. Turner NM. Advanced paediatric life support : de Nederlandse editie. Vijfde druk. ed. Houten :: Bohn Stafleu van Loghum; 2017.
25. Zachariasse JM, Seiger N, Rood PP, Alves CF, Freitas P, Smit FJ, et al. Validity of the Manchester Triage System in emergency care: A prospective observational study. PLoS One. 2017;12(2):e0170811.
26. Lee JY, Oh SH, Peck EH, Lee JM, Park KN, Kim SH, et al. The validity of the Canadian Triage and Acuity Scale in predicting resource utilization and the need for immediate life-saving interventions in elderly emergency department patients. Scand J Trauma Resusc Emerg Med. 2011 Nov 3;19:68.
27. Donders AR, van der Heijden GJ, Stijnen T, Moons KG. Review: a gentle introduction to imputation of missing values. J Clin Epidemiol. 2006 Oct;59(10):1087-91.
28. Sterne JA, White IR, Carlin JB, Spratt M, Royston P, Kenward MG, et al. Multiple imputation for missing data in epidemiological and clinical research: potential and pitfalls. Bmj. 2009 Jun 29;338:b2393.
29. Neuman MI, Chiang VW. Variation in pediatric care at US hospitals. Pediatrics. 2013 Aug;132(2):369-70.
30. Pitts SR, Morgan SR, Schrager JD, Berger TJ. Emergency department resource use by supervised residents vs attending physicians alone. Jama. 2014 Dec 10;312(22):2394-400.
31. White AL, Armstrong PA, Thakore S. Impact of senior clinical review on patient disposition from the emergency department. Emerg Med J. 2010 Apr;27(4):262-5, 96.
32. Hampers LC, Faries SG. Practice variation in the emergency management of croup. Pediatrics. 2002 Mar;109(3):505-8.
33. Melo MR, Ferreira-Magalhaes M, Flor-Lima F, Rodrigues M, Severo M, Almeida-Santos L, et al. Dedicated Pediatricians in Emergency Department: Shorter Waiting Times and Lower Costs. PLoS One. 2016;11(8):e0161149.
34. Knapp JF, Simon SD, Sharma V. Variation and trends in ED use of radiographs for asthma, bronchiolitis, and croup in children. Pediatrics. 2013 Aug;132(2):245-52.
35. Scott-Jupp R, Carter E, Brown N. Effects of consultant residence out-of-hours on acute paediatric admissions. Arch Dis Child. 2020 Jan 14.

Confidential: For Review Only

36. Fullerton K, Depinet H, Iyer S, Hall M, Herr S, Morton I, et al. Association of Hospital Resources and Imaging Choice for Appendicitis in Pediatric Emergency Departments. Acad Emerg Med. 2017 Apr;24(4):400-9.
37. Kharbanda AB, Hall M, Shah SS, Freedman SB, Mistry RD, Macias CG, et al. Variation in resource utilization across a national sample of pediatric emergency departments. J Pediatr. 2013 Jul;163(1):230-6.
38. Florin TA, French B, Zorc JJ, Alpern ER, Shah SS. Variation in emergency department diagnostic testing and disposition outcomes in pneumonia. Pediatrics. 2013 Aug;132(2):237-44.
39. Bourgeois FT, Monuteaux MC, Stack AM, Neuman MI. Variation in emergency department admission rates in US children's hospitals. Pediatrics. 2014 Sep;134(3):539-45.
40. El Khoury AC, Durden E, Ma L, Markson LE, Lee AW, Duan Y, et al. Perception and management of fever in infants up to six months of age: a survey of US pediatricans. BMC Pediatr. 2010 Dec 22;10:95.
41. Pachter LM, Ludwig S, Groves A. Night people: utilization of a pediatric emergency department during the late night. Pediatr Emerg Care. 1991 Feb;7(1):12-4.
42. Duvald I, Moellekaer A, Boysen MA, Vest-Hansen B. Linking the severity of illness and the weekend effect: a cohort study examining emergency department visits. Scand J Trauma Resusc Emerg Med. 2018 Sep 5;26(1):72.
43. Lopez E, Udaondo J, Olabarri M, Martinez-Indart L, Benito J, Mintegi S, et al. Mortality in Spanish pediatric emergency departments: a 5-year multicenter survey. Eur J Emerg Med. 2017 Dec;24(6):392-7.
44. O'Malley P, Barata I, Snow S, American Academy of Pediatrics Committee on Pediatric Emergency M, American College of Emergency Physicians Pediatric Emergency Medicine C, Emergency Nurses Association Pediatric C. Death of a child in the emergency department. Pediatrics. 2014 Jul;134(1):e313-30.
45. van de Maat J, Jonkman H, van de Voort E, Mintegi S, Gervaix A, Bressan S, et al. Measuring vital signs in children with fever at the emergency department: an observational study on adherence to the NICE recommendations in Europe. Eur J Pediatr. 2020 Feb 8.
46. Gravel J, Opatrny L, Gouin S. High rate of missing vital signs data at triage in a paediatric emergency department. Paediatr Child Health. 2006 Apr;11(4):211-5.
47. Seiger N, van Veen M, Almeida H, Steyerberg EW, van Meurs AH, Carneiro R, et al. Improving the Manchester Triage System for pediatric emergency care: an international multicenter study. PLoS One. 2014;9(1):e83267.

Confidential: For Review Only

Table 1. Missing data for vital signs and imputation model[a]

Vital sign	Missing<br>% of total patients (inter-hospital range)
**Heartrate**	42.7 (19.5 – 62.9)
**Respiratory rate**	52.4 (23.5 – 87.4)
**Oxygen saturation**	44.9 (19.7 – 66.4)
**Temperature**	22.5 (5.9 – 58.2)

[a]Missing data for vital signs were imputed using a multiple imputation model including patient characteristics, date and time, triage items, vital signs, diagnostics, therapy and disposition after the ED. This imputation process resulted in twenty-five datasets on which statistical analyses were performed and pooled for a final result.(27) Imputation was performed by using the MICE imputation package in R, version 2.15.2.

Table 2. Patient characteristics

(Total, n=119,204)	**Office hours[a]** **(n=50,417)**	**Evening shift** **(n=36,429)**	**Night shift** **(n=14,138)**	**Day shift weekend** **(n=18,220)**
**Median age, yrs *(IQR[b])***	4.8 (1.8 – 9.9)	4.5 (1.8 – 9.5)	3.7 (1.4 – 8.2)	4.2 (1.7 – 8.8)
**Gender, *n (%)***				
- **Male**	27,150 (53.9)	19,698 (54.1)	7,566 (53.5)	9,956 (54.6)
**MTS, *n (%)***				
- **High urgent**	4,963 (9.9)	4,136 (10.7)	2,302 (16.3)	1,683 (9.2)
- **Urgent**	13,300 (26.4)	10,796 (29.6)	3,655 (25.9)	4,866 (26.7)
- **Low urgent**	31,478 (61.7)	21,276 (58.4)	7,892 (55.8)	11,366 (62.4)
- **Missing**	676 (1.3)	452 (1.2)	289 (2.0)	305 (1.7)
**Vital signs, *n (%)***				
- **Normal**	18,734 (37.2)	12,903 (35.4)	4,812 (34.0)	6,553 (36.0)
- **1 Abnormal**	22,545 (44.7)	15,947 (43.8)	6,100 (43.1)	8,119 (44.6)
- **2 or more abnormal**	9,139 (18.1)	7,578 (20.8)	3,227 (22.8)	3,548 (19.5)

[a]Office hours = Day shift week
[b]IQR = Interquartile range
[c]MTS = Manchester Triage System

For peer review only

Table 3. Management (diagnostics, treatment, hospital admission and PICU admission), descriptive

Total, n = 119,204	Office hours[a] (n=50,417)	Evening shift (n=36,429)	Night shift (n=14,138)	Day shift weekend (n=18,220)
**Mean % (inter-hospital range)**				
**Laboratory diagnostics**				
**Any lab test (n=119,204)**	22.2 (11.7 – 40.6)	18.6 (9.2 – 33.0)	21.5 (9.8 – 40.0	20.2 (8.0 – 33.3)
**Urine tests (n=50,473)** [b]	20.2 (11.0 - 23.5)	17.1 (8.1 – 22.3)	20.2 (13.9 – 23.2)	16.6 (7.7 – 19.6)
**Blood tests (n=66,029)**[b]	21.9 (11.7 – 26.7)	15.2 (9.2 – 20.3)	20.4 (9.8 - 30.5)	17.2 (7.9 – 23.5)
**Imaging diagnostics**				
**X-ray (n=119,204)**	19.5 (6.0 - 24.7)	19.4 (3.7 – 40.9)	12.1 (4.3 – 17.1)	17.4 (5.5 -41.2)
**Ultrasound (n=119,204)**	2.7 (1.5 - 6.0)	1.7 (0.5 - 2.9)	1.2 (0.5 - 4.1)	2.2 (0.6 - 4.2)
**CT (n=119,204)**	1.2 (0.7 - 2.5)	1.0 (0.6 - 2.4)	0.9 (0.4 - 2.3)	1.0 (0.5 - 2.1)
**Treatment**				
**Oral medication (n=108,621)**[b]	13.9 (8.0 – 34.3)	17.2 (8.6 – 41.8)	23.5 (14.9 – 45.1)	16.5 (8.2 – 39.7)
**Inhaled medication (n=119,204)**	6.5 (3.6 - 8.7)	6.5 (3.5 – 9.0)	12.0 (6.0 – 15.4)	6.9 (3.5 – 9.8)
**IV[c] medication or fluids (n=119,204)**	7.3 (4.1 - 14.0)	7.0 (4.2 - 11.3)	11.3 (4.6 - 19.4)	7.8 (3.4 - 12.0)
**ILI[d] (n=119,204)**	0.3 (0.0 – 1.1)	0.3 (0.0 -1.2)	0.5 (0.0 -1.9)	0.4 (0.1 -1.2)
**Hospital admission (n=119,204)**				
**Yes**	9.7 (4.3 - 22.6)	10.2 (5.0 – 24.2)	14.3 (8.8 – 38.4)	9.9 (4.6 – 22.4)
**PICU admission (n=119,204)**				
**Yes**	0.6 (0.1 – 2.3)	0.5 (0.1 -2.8)	0.8 (0.2 - 4.7)	0.6 (0.0 - 2.7)

[a]Office hours = Day shift week
[b]Not all variables were complete for all hospitals, making the total number less than 119.204.
[c] IV = Intravenous
[d] ILI = Immediate Lifesaving Interventions

Confidential: For Review Only

Table 4. The association between management (lab, imaging, treatment, hospital and PICU[a] admission) and time of arrival at the ED, adjusted regression analyses

Total, n = 119,204 aOR[b] (95% CI)	**Office hours[c] (n=50417)**	**Evening shift (n=36,429)**	**Night shift (n=14,138)**	**Day shift weekend (n=18,220)**
**Lab**	- -	0.79 (0.76 – 0.82) *	0.82 (0.78 – 0.86) *	0.92 (0.87 – 0.96) *
**Imaging**	- -	0.93 (0.89 – 0.96) *	0.64 (0.60 – 0.67) *	0.99 (0.94 – 1.04)
**Treatment**	- -	1.11 (1.07 – 1.15) *	1.56 (1.49 – 1.63) *	1.17 (1.12 – 1.22) *
**Hospital admission**	- -	1.06 (1.00 – 1.11) *	1.32 (1.24 – 1.41) *	1.05 (0.99 – 1.12)
**PICU[a] admission**	- -	1.15 (0.95 – 1.42)	0.92 (0.71 – 1.19)	1.19 (0.93 – 1.52)

[a] PICU = Paediatric Intensive Care Unit
[b]aOR = Adjusted Odds Ratio. Adjusted for age, gender, urgency, vital signs, presenting problem and hospital.
[c] Office hours (= day shift week) as reference shift.
* P-value ≤0.05. If not stated: p-value >0.05

Confidential: For Review Only

Table 5. Hospital characteristics

	Erasmus MC - Sophia Children's Hospital, Rotterdam the Netherlands	Maasstad Hospital, Rotterdam, the Netherlands	St. Mary's hospital Imperial College Healthcare NHS Trust, London, United Kingdom	Hospital Prof. Dr. Fernando Fonseca, Lisbon, Portugal	General Hospital, Vienna, Austria
**Hospital characteristics**	University hospital<br>60 paediatric beds	Teaching hospital<br>59 paediatric beds	University hospital<br>46 paediatric beds	Community hospital<br>91 paediatric beds	University hospital<br>134 paediatric beds
**Emergency department characteristics[a]**	6,500 children/year<br><br>Urban<br><br>Mixed high and low socio-economic status	9,500 children/year<br><br>Urban<br><br>Generally low socio-economic status	27,000 children/year<br><br>Urban<br><br>Mixed high and low socio-economic status	60,000 children/year<br><br>Mixed urban/rural<br>Generally low socio-economic status	22,000 children/year<br><br>Urban<br><br>Mixed high and low socio-economic status
**Number of patients included**	18,590	10,583	15,556	53,175	21,300
**Inclusion period**	01-01-2012 to 31-12-2014	01-05-2014 to 31-10-2015	01-07-2014 to 28-02-2015	01-03-2014 to 28-02-2015	01-01-2014 to 31-12-2014
**Number of patients during:**<br>**- Office hours**<br>**- Out-of-office hours**	 8,915 (48.0%)<br>9,675 (52.0%)	 4,229 (40.0%)<br>6,354 (60.0%)	 5,635 (36.2%)<br>9,921 (63.8%)	 22,470 (42.3%)<br>30,705 (57.7%)	 9,168 (43.0%)<br>12,132 (57.0%)
**Median age,** ***yrs (IQR[b])***	4.3 (1.4 – 9.8)	5.7 (1.9 – 11.6)	3.9 (1.5 – 8.8)	4.7 (1.9 – 9.5)	3.9 (1.6 – 8.3)
**MTS,** ***n (%)***					
- **High urgent**	2,427 (13.1)	1,515 (14.3)	1,605 (10.3)	6,222 (11.7)	1,084 (5.0)
- **Urgent**	8,744 (47.0)	5,110 (48.3)	3,961 (25.5)	10,951 (20.6)	3851 (18.1)
- **Low urgent**	6,849 (36.8)	3,857 (36.4)	9,990 (64.2)	36,002 (67.7)	15,314 (71.9)
- **Missing**	570 (3.1)	101 (0.1)	0 (0.0)	0 (0.0)	1,051 (4.9)
**Presenting problem,** ***n (%)***					
- **Medical**	12.206 (65.7)	5,163 (48.8)	11.520 (74.1)	43,814 (82.4)	19,174 (90.0)
- **Trauma**	5,814 (31.3)	5,321 (50.3)	4,036 (25.9)	9,361 (17.6)	1,034 (4.9)
- **Missing**	570 (3.1)	99 (0.9)	0 (0.0)	0 (0.0)	1,092 (5.1)

[a]All emergency departments (ED) are paediatric only, except for the Maasstad Hospital, which is mixed adult-paediatric. The Erasmus MC-Sophia Children's hospital has a mixed adult-paediatric ED since October 2014 (3 months in total during the inclusion period).
[b]IQR = Interquartile range.

Confidential: For Review Only

Figure 1. Presenting problems

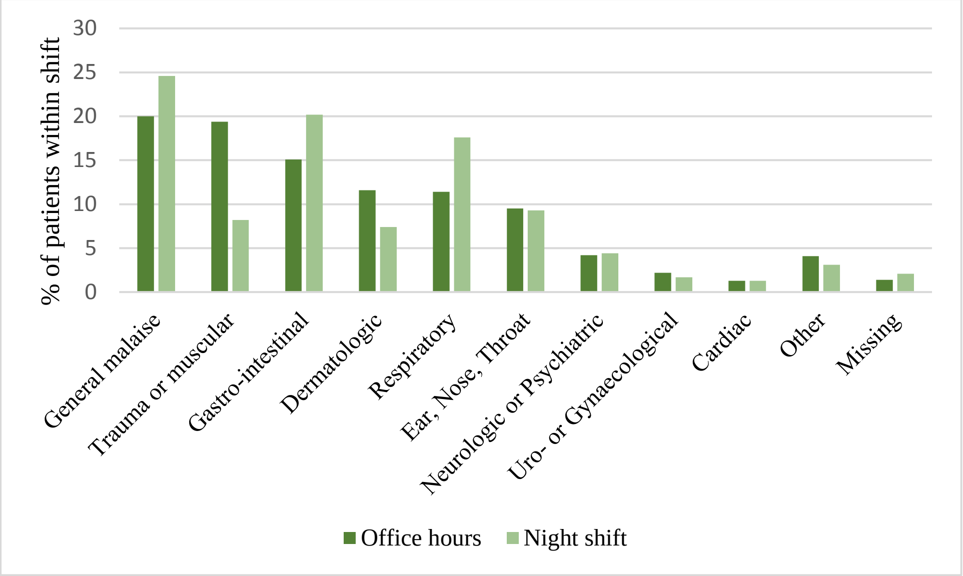

tial: For Review Only

SUPPLEMENTARY APPENDIX

Supplement 1. Presenting problems

Supplement 2. Immediate Life-saving Interventions

Supplement 3. Missing data for vital signs

Supplement 4. Complete case analyses

Supplement 5. Hospital characteristics

Supplement 6. Stratified regression analyses

Confidential: For Review Only

Supplement 1. Presenting problems

Supplementary table 1.1 Presenting problems definitions	
**Subgroups[a]**	**MTS Flowchart**
**General malaise**	Crying baby. Unwell child. Worried parent. Irritable child. Unwell adult.
**Trauma or muscular**	Assault. Falls. Head injury. Limb problems. Major trauma. Torso injury. Neck pain. Back pain. Limping child.
**Gastro-intestinal**	Abdominal pain in adults. Abdominal pain in children. Diarrhoea and vomiting. GI Bleeding.
**Respiratory**	Asthma. Shortness of breath in adults. Shortness of breath in children.
**Eear, Nose, Throat**	Ear problems. Sore throat. Facial problems.
**Neurologic or Psychiatric**	Behaving strangely. Fits. Headache. Apparently drunk. Collapsed adult. Overdose and poisoning. Self-harm. Mental illness.
**Dermatologic**	Bites and stings. Burns and scalds. Wounds. Abscesses and local infections. Rashes.
**Uro- or Gynaecological**	Testicular pain. Urinary problems. Sexually acquired infection. Pregnancy. PV Bleeding.
**Cardiac**	Chest pain. Palpitations.
**Other**	Allergy. Dental problems. Diabetes. Exposure to chemicals. Eye problems. Foreign body. Major incidents – primary. Major incidents – secondary. General/other.

[a]Subgroups of presenting problems (MTS Flowchart), based on a previous study (1)

Supplementary table 1.2 Presenting problems				
**N (%)**	**Office hours (n=50,417)**	**Evening shift (n=36,429)**	**Night shift (n=14,138)**	**Day shift weekend (n=18,220)**
**General malaise**	10,069 (20.0)	7,021 (19.3)	3,473 (24.6)	3,784 (20.8)
**Trauma or muscular**	9,785 (19.4)	7,281 (20.0)	1,161 (8.2)	3,111 (17.1)
**Gastro-intestinal**	7,613 (15.1)	5,274 (14.5)	2,860 (20.2)	2,470 (13.6)
**Dermatologic**	5,864 (11.6)	4,851 (13.3)	1,044 (7.4)	2,864 (15.7)
**Respiratory**	5,686 (11.3)	3,833 (10.5)	2,494 (17.6)	1,947 (10.7)
**Ear, Nose, Throat**	4,777 (9.5)	3,596 (9.9)	1,320 (9.3)	1,870 (10.3)
**Neurologic or Psychiatric**	2,115 (4.2)	1,384 (3.8)	628 (4.4)	566 (3.1)
**Uro- or Gynaecological**	1,126 (2.2)	800 (2.2)	240 (1.7)	416 (2.3)
**Cardiac**	635 (1.3)	438 (1.2)	177 (1.3)	148 (0.8)
**Other**	2,084 (4.1)	1,489 (4.1)	443 (3.1)	733 (4.0)
**Missing**	690 (1.4)	462 (1.3)	298 (2.1)	311 (1.7)

Confidential: For Review Only

Supplement 2. Immediate life-saving interventions

Supplementary table 2. Immediate life-saving interventions[a]
1. Airway and breathing support, including intubation or emergent non-invasive positive pressure ventilation.
2. Electrical therapy, including defibrillation, emergent cardioversion, or external pacing.
3. Procedures, including chest needle decompression, pericardiocentesis, or open thoracotomy.
4. Hemodynamic support, including significant intravenous fluid resuscitation in the setting of hypotension, blood administration, or control of major bleeding.
5. Emergency medications, including naloxone, dextrose, atropine, adenosine, epinephrine, or vasopressors

[a]Adapted from Lee et. al.(2)

Confidential: For Review Only

Supplement 3. Missing data for vital signs

The missing data for the vital signs variables (heart rate 42.7%, respiratory rate 52.4%, oxygen saturation 44.9%, temperature 22.5%) were assumed to be missing at random. We expected that the pattern of missingness could be explained by different patient and setting-related factors, that were included in the dataset. For example, based on clinical experience, measuring vital signs was considered less useful or relevant in low urgency cases or in patients with specific presenting problems, such as simple fractures. Also in high urgency cases data of vital signs was missing, which was not expected. This could be partly explained by data of vital signs that were not documented in the patient record, instead of vital signs that were not measured. The proportion of missing data for vital signs was comparable with previous studies. (3-5) A variety in the proportion of missingness was observed between the different hospitals, which is likely due to differences in characteristics of the patient populations and in local policies and compliance to these policies. (Supplementary figure 1)
In exploratory analyses, strong associations were found between the proportion of missing data and several patient and hospital-related factors (type of presenting complaint, triage urgency and disposition after the ED visit), thereby supporting the assumption that the missing data for vital signs were missing at random.
The missing data were imputed using a multiple imputation model including general patient characteristics, date and time of visit, triage items, vital signs, diagnostics, therapy and disposition. (Supplementary table 3)
Imputation was performed by using the MICE imputation package in R, version 2.15.2. The imputation process resulted in twenty-five datasets on which statistical analyses were performed and pooled for a final result.

Supplementary table 3. Multiple imputation model

**Type of information**	**Variables**
**General patient characteristics**	Hospital (cat); Age (cont.); Gender (M/F); Referral type (Self / GP / Emergency services / Other)
**Date and time**	Arrival month (1-12); Arrival hour (morning/evening/night); Arrival day (week/weekend)
**Triage items**	MTS flowchart (cat); MTS urgency (1-5); MTS painscore (cont)
**Physiological parameters**	Heart rate (cont); Respiratory rate (cont); Temperature (cont); Systolic bloodpressure (cont); Diastolic bloodpressure (cont); Saturation (cont); Capillary refill (dich); Consciousness (normal / decreased / unconsciousness);
**Diagnostics, Therapy, Disposition**	Any lab (Yes/No); Any imaging (Yes/No); Any cultures (Yes/No); Oxygen therapy (Yes/No); Oral meds (Yes/No); Inhalation meds (Yes/No); IV meds or fluids (Yes/No); Immediate lifesaving interventions (Yes/No); Disposition (Mortality or ICU admission/ Admission / Other (cat)

Confidential: For Review Only

Supplementary figure 1. Missing data for the vital signs variables

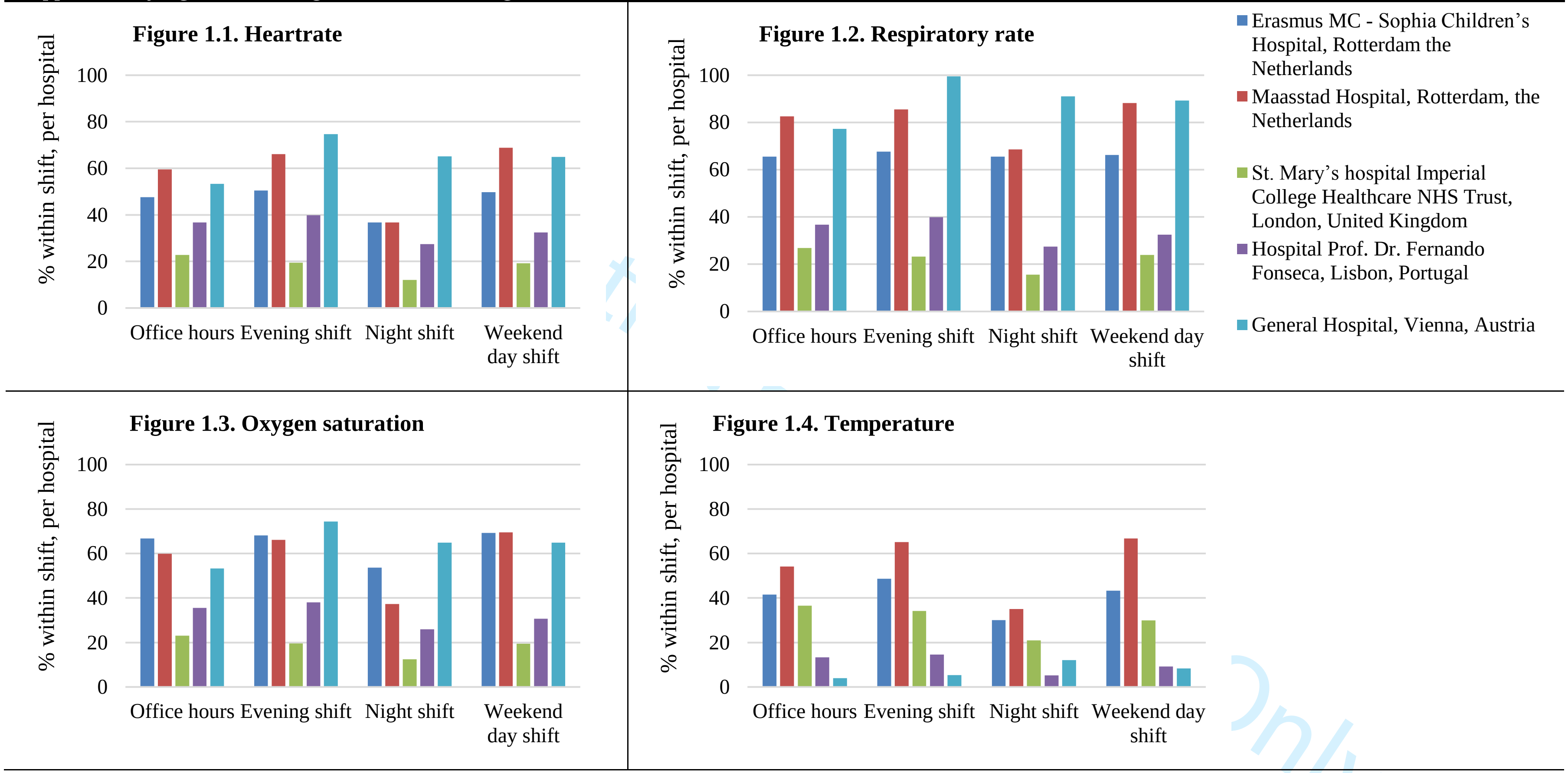

Supplement 4. Complete case analyses

For the complete case analyses, we used a database consisting only of cases in which all vital signs (heart rate, respiratory rate, oxygen saturation and temperature) were measured and documented (total n=51,664). We performed the same regression analyses as with the imputation database, adjusted for age, gender, urgency, vital signs, presenting problem and hospital.
We found comparable results of the association between management and time of arrival at the ED, when comparing the results of the imputation dataset to the complete case dataset (Supplementary table 4). In the complete case analyses, imaging was less likely to be requested and treatment was more likely to be given during all out-of-office hours. During the night patients were significantly more likely to be admitted to the hospital. In complete case analyses, only the slightly lower number of laboratory diagnostics and slightly higher number of hospital admissions was not seen in weekend day shifts, but was confirmed in evening shifts. More chance variation in complete case analyses is likely to contribute to these differences. (6, 7)

Supplementary table 4 The association between management (lab, imaging, treatment, hospital and PICU[a] admission) and time of arrival at the ED, adjusted regression analyses. Complete cases (vital signs).

Total, n = 51,664<br>aOR[b] (95% CI)	**Office hours[c] (n=21,963)**	**Evening shift (n=7,800)**	**Night shift (n=14,251)**	**Day shift weekend (n=7,649)**
**Lab**	-<br>-	0.95<br>(0.89 – 1.00)	0.88<br>(0.81 – 0.95) *	1.02<br>(0.95 – 1.10)
**Imaging**	-<br>-	0.85<br>(0.80 – 0.91) *	0.63<br>(0.58 – 0.68) *	0.92<br>(0.85 – 0.99)*
**Treatment**	-<br>-	1.11<br>(1.05 – 1.17) *	1.46<br>(1.37 – 1.55) *	1.17<br>(1.10 – 1.24) *
**Hospital admission**	-<br>-	1.05<br>(0.97 – 1.14)	1.27<br>(1.16 – 1.39) *	0.95<br>(0.86 – 1.05)

[a] PICU = Paediatric Intensive Care Unit
[b]aOR = Adjusted Odds Ratio. Adjusted for age, gender, urgency, vital signs, presenting problem and hospital.
[c] Office hours (= day shift week) as reference shift.
* P-value ≤0.05. If not stated: p-value >0.05

Confidential: For Review Only

Supplement 5. Hospital characteristics

Supplementary table 5.1 Patient characteristics - Erasmus MC - Sophia Children's Hospital, Rotterdam the Netherlands

(Total, n=18,590)	**Office hours[a]** **(n=8,915)**	**Evening shift** **(n=5,055)**	**Night shift** **(n=1,618)**	**Day shift weekend** **(n=3,002)**
**Median age, yrs *(IQR[b])***	4.6 (1.4 – 10.2)	4.1 (1.4 – 9.4)	3.7 (1.2 – 9.3)	4.2 (1.5 – 9.2)
**Gender, *n (%)***				
- **Male**	5,098 (57.2)	2,956 (58,5)	957 (59,1)	1,760 (58,6)
**MTS, *n (%)***				
- **High urgent**	1,073 (12.0)	655 (12.9)	356 (22.0)	343 (11.4)
- **Urgent**	4,040 (45.3)	2,502 (49.5)	741 (45.8)	1,461 (48.7)
- **Low urgent**	3,462 (38.8)	1,762 (34,8)	492 (30.4)	1,133 (37.7)
- **Missing**	340 (3.8)	136 (2.7)	29 (1.8)	65 (2.2)
**Vital signs, *n (%)***				
- **Normal**	2,925 (32.8)	1,559 (30.9)	440 (27.2)	962 (32.1)
- **1 Abnormal**	3,885(43.6)	2,114 (41.8)	681 (42.1)	1,289 (42.9)
- **2 or more abnormal**	2,105 (23.6)	1,381 (27.3)	497 (30.7)	751 (25.0)

[a]Office hours = Day shift week
[b]IQR = Interquartile range
[c]MTS = Manchester Triage System

Supplementary table 5.2 Patient characteristics - Maasstad Hospital, Rotterdam, the Netherlands

(Total, n=10,583)	**Office hours[a]** **(n=4,229)**	**Evening shift** **(n=3,630)**	**Night shift** **(n=981)**	**Day shift weekend** **(n=1,743)**
**Median age, yrs *(IQR[b])***	5.5 (1.8 – 11.4)	6.2 (2.2 – 11.7)	3.8 (1.0 – 10.5)	6.7 (2.5 – 11.9)
**Gender, *n (%)***				
- **Male**	2,384 (56.4)	1,999 (55.1)	573 (58.4)	1,048 (60.1)
**MTS, *n (%)***				
- **High urgent**	586 (13.9)	477 (13.2)	255 (26.0)	197 (11.3)
- **Urgent**	2,023 (47.8)	1,788 (49.3)	449 (45.8)	850 (48.8)
- **Low urgent**	1,567 (37.1)	1,339 (36.9)	267 (27.2)	684 (39.2)
- **Missing**	53 (1.3)	26 (0.7)	10 (1.0)	12 (0.7)
**Vital signs, *n (%)***				
- **Normal**	1,305 (30.9)	1,076 (29.7)	250 (25.5)	540 (31.0)
- **1 Abnormal**	1,839 (43.5)	1,526 (42.0)	387 (39.4)	747 (42.9)
- **2 or more abnormal**	1,085 (25.6)	1,028 (28.3)	344 (35.1)	456 (26.1)

[a]Office hours = Day shift week
[b]IQR = Interquartile range
[c]MTS = Manchester Triage System

For peer review only

Supplementary table 5.3 Patient characteristics - St. Mary's hospital Imperial College Healthcare NHS Trust, London, United Kingdom				
(Total, n=15,556)	**Office hours[a] (n=5,635)**	**Evening shift (n=4,847)**	**Night shift (n=2,429)**	**Day shift weekend (n=2,645)**
**Median age, yrs *(IQR[b])***	4.3 (1.5 – 9.6)	4.0 (1.5 – 8.9)	3.4 (1.3 – 7.4)	3.7 (1.5 – 7.7)
**Gender, *n (%)***				
- **Male**	3,137 (55.7)	2,736 (56.4)	1,328 (54.7)	1,476 (55.8)
**MTS, *n (%)***				
- **High urgent**	567 (10.1)	494 (10.2)	312 (12.8)	232 (8.7)
- **Urgent**	1,456 (25.8)	1,318 (27.2)	568 (23.4)	619 (23.4)
- **Low urgent**	3,612 (64.1)	3,035 (62.6)	1,549 (63.8)	1,794 (67.9)
- **Missing**	0 (0.0)	0 (0.0)	0 (0)	0 (0)
**Vital signs, *n (%)***				
- **Normal**	2,494 (44.3)	2,138 (44.1)	1,008 (41.5)	1,167 (44.1)
- **1 Abnormal**	2,152 (38.2)	1,801 (37.2)	887 (36.5)	1,010 (38.2)
- **2 or more abnormal**	989 (17.5)	909 (18.7)	534 (22.0)	468 (17.7)

[a]Office hours = Day shift week
[b]IQR = Interquartile range
[c]MTS = Manchester Triage System

Supplementary table 5.4 Patient characteristics - Hospital Prof. Dr. Fernando Fonseca, Lisbon, Portugal				
(Total, n=53,175)	**Office hours[a] (n=22,470)**	**Evening shift (n=16,422)**	**Night shift (n=6,879)**	**Day shift weekend (n=7,404)**
**Median age, yrs *(IQR[b])***	5.2 (2.1 – 10.1)	4.7 (2.0 – 9.4)	3.8 (1.5 – 7.9)	4.3 (1.9 – 8.5)
**Gender, *n (%)***				
- **Male**	11,765 (52.4)	8,567 (52.2)	3,513 (51.1)	3,840 (51.9)
**MTS, *n (%)***				
- **High urgent**	2,310 (10.3)	1,935 (11.8)	1,191 (17.8)	756 (10.2)
- **Urgent**	4,281 (19.1)	3,844 (23.4)	1,445 (21.0)	1,381 (18.7)
- **Low urgent**	15,879 (70.6)	10,643 (64.8)	4,213 (61,3)	5,267 (71.1)
- **Missing**	0 (0.0)	0 (0.0)	0 (0.0)	0 (0.0)
**Vital signs, *n (%)***				
- **Normal**	8,740 (38.9)	5,924 (36.1)	2,374 (34.5)	2,700 (36.5)
- **1 Abnormal**	10,435 (46.4)	7,608 (46.3)	3,145 (45.7)	3,500 (47.3)
- **2 or more abnormal**	3,295 (14.7)	2,890 (17.6)	1,359 (19.8)	1,204 (16.2)

[a]Office hours = Day shift week
[b]IQR = Interquartile range
[c]MTS = Manchester Triage System

For peer review only

Supplementary table 5.5 Patient characteristics – General Hospital, Vienna, Austria				
(Total, n=21,300)	**Office hours[a] (n=9,168)**	**Evening shift (n=6,475)**	**Night shift (n=2,231)**	**Day shift weekend (n=3,426)**
**Median age, yrs (*IQR[b]*)**	4.0 (1.6 – 8.5)	3.8 (1.5 – 8.4)	3.7 (1.4 – 8.3)	3.8 (1.7 – 7.6)
**Gender, *n (%)***				
- **Male**	4,766 (52.0)	3,440 (53.1)	1,195 (53.6)	1,832 (53.5)
**MTS, *n (%)***				
- **High urgent**	427 (4.6)	343 (5.3)	158 (7.1)	155 (4.5)
- **Urgent**	1,500 (16.4)	1,344 (20.8)	452 (20.3)	555 (16.2)
- **Low urgent**	6,958 (75.9)	4,297 (69.5)	1,371 (61.4)	2,488 (72.6)
- **Missing**	283 (3.1)	290 (4.5)	250 (11.2)	228 (6.7)
**Vital signs, *n (%)***				
- **Normal**	3,271 (35.7)	2,206 (34.1)	739 (33.1)	1,183 (34.5)
- **1 Abnormal**	4,233 (46.2)	2,899 (44.7)	1,000 (44.8)	1,573 (45.9)
- **2 or more abnormal**	1,664 (18.1)	1,371 (21.2)	493 (22.1)	670 (19.6)

[a]Office hours = Day shift week
[b]IQR = Interquartile range
[c]MTS = Manchester Triage System

Confidential: For Review Only

Supplement 6. Stratified regression analyses

Supplementary table 6. Management adjusted regression analyses, per hospital

Total n=119,204 aOR[a] (95% CI)	Erasmus MC - Sophia Children's Hospital, Rotterdam the Netherlands n = 18,590	Maasstad Hospital, Rotterdam, the Netherlands n = 10,583	St. Mary's hospital Imperial College Healthcare NHS Trust, London, United Kingdom n = 15,556	Hospital Prof. Dr. Fernando Fonseca, Lisbon, Portugal n = 53,175	General Hospital, Vienna, Austria n = 21,300
**Lab**					
- Evening shift	0.75 (0.69 – 0.82) *	0.82 (0.71 – 0.94) *	0.73 (0.63 – 0.83)*	1.00 (0.94 – 1.07)	0.61 (0.57 – 0.66) *
- Night shift	0.77 (0.68 – 0.87) *	0.86 (0.71 – 1.04)	0.71 (0.60 – 0.84) *	1.03 (0.95 – 1.11)	0.68 (0.61 – 0.77) *
- Day shift weekend	0.90 (0.81 – 0.99)*	0.85 (0.71 – 1.02)	0.68 (0.57 – 0.81)*	1.15 (1.07 – 1.25) *	0.82 (0.74 – 0.90) *
**Imaging**					
- Evening shift	0.73 (0.67 – 0.80) *	2.18 (1.93 – 2.47) *	0.77 (0.68 – 0.87) *	0.94 (0.88 – 0.99) *	0.59 (0.52 – 0.68) *
- Night shift	0.53 (0.46 – 0.62)*	1.05 0.84 – 1.30	0.52 (0.43 – 0.62) *	0.68 0.63 – 0.74 *	0.55 (0.44 – 0.67)*
- Day shift weekend	0.85 (0.76 – 0.94)	2.00 (1.72 – 2.34) *	0.81 (0.69 – 0.94) *	0.94 (0.88 – 1.02)	1.00 (0.86 – 1.16)
**Treatment**					
- Evening shift	1.13 (1.04 – 1.22) *	0.78 0.68 – 0.89) *	1.35 (1.25 – 1.47)*	0.99 (0.93 – 1.04)	1.44 (1.32 – 1.58) *
- Night shift	1.32 (1.17 – 1.50)*	1.03 (0.86 – 1.24)	1.55 (1.40 – 1.72) *	1.53 (1.43 – 1.64) *	2.32 (2.06 – 2.62) *
- Day shift weekend	1.12 1.01 – 1.23*	0.89 (0.75 – 1.06)	1.31 (1.19 – 1.54) *	1.12 (1.03 – 1.21) *	1.38 (1.23 – 1.55) *
**Hospital admission**					
- Evening shift	1.20 (1.09 – 1.31) *	1.05 (0.93 – 1.18)	0.92 (0.80 – 1.06)	1.05 (0.95 – 1.16)	0.93 (0.80 – 1.09)
- Night shift	1.43 (1.25 – 1.63) *	1.44 (1.22 – 1.71) *	0.95 (0.80 – 1.19)	1.51 (1.35 – 1.69) *	1.06 (0.86 – 1.30)
- Day shift weekend	1.15 (1.03 – 1.29 *	1.04 (0.89 – 1.21)	0.71 (0.59 – 0.85)*	1.09 (0.95 – 1.24)	1.32 (1.10 – 1.58) *

[a]aOR = Adjusted Odds Ratio. Adjusted for age, gender, urgency level, abnormal vital signs, presenting problem
*P-value ≤0.05. If not stated: p-value >0.05

For peer review only

1. Zachariasse JM, Seiger N, Rood PP, Alves CF, Freitas P, Smit FJ, et al. Validity of the Manchester Triage System in emergency care: A prospective observational study. PLoS One. 2017;12(2):e0170811.
2. Lee JY, Oh SH, Peck EH, Lee JM, Park KN, Kim SH, et al. The validity of the Canadian Triage and Acuity Scale in predicting resource utilization and the need for immediate life-saving interventions in elderly emergency department patients. Scand J Trauma Resusc Emerg Med. 2011 Nov 3;19:68.
3. van de Maat J, Jonkman H, van de Voort E, Mintegi S, Gervaix A, Bressan S, et al. Measuring vital signs in children with fever at the emergency department: an observational study on adherence to the NICE recommendations in Europe. Eur J Pediatr. 2020 Feb 8.
4. Gravel J, Opatrny L, Gouin S. High rate of missing vital signs data at triage in a paediatric emergency department. Paediatr Child Health. 2006 Apr;11(4):211-5.
5. Seiger N, van Veen M, Almeida H, Steyerberg EW, van Meurs AH, Carneiro R, et al. Improving the Manchester Triage System for pediatric emergency care: an international multicenter study. PLoS One. 2014;9(1):e83267.
6. Donders AR, van der Heijden GJ, Stijnen T, Moons KG. Review: a gentle introduction to imputation of missing values. J Clin Epidemiol. 2006 Oct;59(10):1087-91.
7. Sterne JA, White IR, Carlin JB, Spratt M, Royston P, Kenward MG, et al. Multiple imputation for missing data in epidemiological and clinical research: potential and pitfalls. Bmj. 2009 Jun 29;338:b2393.

Confidential: For Review Only
